# Single‐cell transcriptome analysis reveals functional changes in tumour‐infiltrating B lymphocytes after chemotherapy in oesophageal squamous cell carcinoma

**DOI:** 10.1002/ctm2.1181

**Published:** 2023-01-17

**Authors:** Shoichi Nakamura, Kenoki Ohuchida, Yoshiki Ohtsubo, Yutaka Yamada, Chikanori Tsutsumi, Sho Okuda, Kyoko Hisano, Yuki Mochida, Tomohiko Shinkawa, Chika Iwamoto, Nobuhiro Torata, Yusuke Mizuuchi, Koji Shindo, Kohei Nakata, Taiki Moriyama, Takehiro Torisu, Eishi Nagai, Takashi Morisaki, Takanari Kitazono, Yoshinao Oda, Masafumi Nakamura

**Affiliations:** ^1^ Department of Surgery and Oncology Graduate School of Medical Sciences Kyushu University Fukuoka Japan; ^2^ Department of Anatomic Pathology Graduate School of Medical Sciences Kyushu University Fukuoka Japan; ^3^ Department of Hematology Clinical Immunology and Infectious Diseases Graduate School of Medicine Ehime University Ehime Japan; ^4^ Department of Medicine and Clinical Science Graduate School of Medical Sciences Kyushu University Fukuoka Japan; ^5^ Department of Surgery Fukuoka Red Cross Hospital Fukuoka Japan; ^6^ Department of Cancer Immunotherapy Fukuoka General Cancer Clinic Fukuoka Japan

**Keywords:** oesophageal squamous cell carcinoma, neoadjuvant chemotherapy, single‐cell transcriptome, tumour immune microenvironment, tumour infiltrating B lymphocytes

## Abstract

**Background:**

Tumour immune microenvironment is related with carcinogenesis and efficacy of immunotherapy. B cells play major roles in humoral immunity, but detailed functions of tumour‐infiltrating B lymphocytes (TIL‐Bs) are unknown. Therefore, our aim was to investigate the functional heterogeneity of TIL‐Bs in oesophageal squamous cell carcinoma (ESCC) and lymph nodes (LNs) during chemotherapy.

**Methods:**

Single‐cell transcriptome analysis was performed on 23 specimens. We also performed immunohistochemical analysis of immunoglobulin κ C (IGKC), an antibody‐secreting cell (ASC) marker, in 166 ESCC samples and evaluated the implication of IGKC in 2‐year recurrence free survival (RFS) and 3‐year overall survival (OS).

**Results:**

A total of 81,246 cells were grouped into 24 clusters. We extracted B cell clusters based on canonical markers and identified 12 TIL‐B subtypes in ESCC. We found that several functions, such as co‐stimulation and CD40 signalling, were enhanced in TIL‐Bs after chemotherapy. The proportion of naive B cells (NBCs) decreased and B cell activation genes were up‐regulated in NBCs after chemotherapy. The proportion of ASCs in tumours increased with the loss of migratory abilities and antibody production in ASCs was promoted after chemotherapy. Differentially expressed genes up‐regulated with chemotherapy in ASCs correlated with prolonged survival with oesophageal cancer (*p* = .028). In a metastatic LN, the ASC proportion increased and B cell differentiation was enhanced. In immunohistochemical analysis, RFS and OS of high IGKC expression cases were significantly better than those of low IGKC expression cases (RFS: *p* < .0001, OS: *p* < .0001). And in multivariable analysis, the expression of IGKC was an independent favourable prognostic factor for RFS (hazard ratio (HR): 0.23, 95% confidence interval (CI): 0.12–0.45, *p* < .0001) and OS (HR: 0.20, 95% CI: 0.086–0.47, *p* = .0002) in ESCC.

**Conclusions:**

Our findings provide novel insights for the heterogeneity of TIL‐Bs during chemotherapy and will be useful to understand the clinical importance of TIL‐Bs.

## Graphical abstract

Single‐cell transcriptome revealed the heterogeneity of TIL‐Bs in ESCC.

NACT activated NBCs and enhanced antibody production in ASCs.

The IGKC expression, an ASC marker, was a favourable prognostic marker in ESCC.

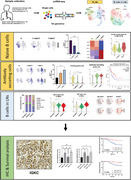

## 1 INTRODUCTION

Oesophageal cancer is the sixth leading cause of cancer‐related mortality in the world, with 544,400 deaths reported in 2020.^1^ Patients with metastatic oesophageal cancer have a poor prognosis, and the 5‐year overall survival (OS) rate of metastatic oesophageal cancer is only 5%.^2^ Oesophageal cancer remains difficult to cure and there is an urgent need to find novel treatments. Recently, a clinical trial for oesophageal squamous cell carcinoma (ESCC) reported that a combination of chemotherapy and immune checkpoint blockage (ICB) was effective for patients with unresectable advanced or recurrent ESCC.^3^ Previous studies reported that ICB response varies depending on the heterogeneity of the tumour immune microenvironment (TIME).^4^ Chemotherapeutic agents induce the increase in the immunogenicity of tumour cells via inducing immunogenic cell death, leading to modification of the TIME.^5^ Therefore, understanding changes in immune heterogeneity during carcinogenesis and chemotherapy is needed to develop new treatments of ESCC patients.

The TIME is a complex structure that consists of various immune cell types, such as T cells, B cells, dendritic cells and macrophages, and each immune cell type has heterogeneity. The TIME is affected by chronic inflammation, carcinogenesis and chemotherapy. Tumour cells obtain tumour‐associated antigens (TAAs), which are generated from mutations in protein‐encoding genes or epigenetic changes during malignant transformation.^6^ In the TIME, T cells, B cells and macrophages require activation to respond to TAAs. T cells and B cells are the most abundant lymphocyte proportions in the TIME and play crucial roles in anti‐tumour immunity. Tumour‐infiltrating T lymphocytes (TIL‐Ts) play central roles in anti‐tumour immunity and have been extensively investigated.^7^ Recently, several studies reported that tumour‐infiltrating B lymphocytes (TIL‐Bs) are related with a good prognosis in several malignant tumours^8^, ^9^ and the efficacy of ICBs.^10^ However, how TIL‐Bs function with TIL‐Ts or other cell types in the TIME remain unclear.

B cells are the main humoral immune cells and secrete large amounts of antibodies. Antibodies against TAAs promote anti‐tumour immunity by inducing antibody‐dependent cellular cytotoxicity (ADCC) and antibody‐dependent cellular phagocytosis (ADCP).^11^ TIL‐Bs secrete several cytokines and chemokines.^12^ Additionally, TIL‐Bs present antigens and promote amplification of TIL‐Ts in non‐small cell lung cancer.^13^ In hepatocellular carcinoma, memory B cells localised in the tumour margin produce granzyme B and IFNγ.^14^ These findings have revealed the heterogeneity of TIL‐B subtypes and functions in the TIME of several cancers. However, how TIL‐B subtypes relate to anti‐tumour immunity in ESCC remains unknown.

On the other hand, lymph nodes (LNs) are the site of drainage of pathogenic and tumour antigens. Complex interactions among T cells, B cells, dendritic cells and other immune cells in LNs provide adaptive immune responses. B cells present antigens to T cells, accumulate activation of T cells in the draining LNs^15^ and secrete specific antibodies against TAAs.^16^ In metastatic LNs, metastatic tumour cells remodel the immune environment to evade the immune system, increase the regulatory T cell proportion and promote CD8^+^ T cell dysfunction.^17^ Nonetheless, the detailed mechanism of the B cell response to TAAs in tumour‐related LNs remains unclear.

Single‐cell RNA sequence (scRNA‐seq) enables researchers to evaluate the heterogeneity of the TIME with high resolution, and many types of tumours have been analysed by scRNA‐seq.^18^, ^19^ The characterisation, heterogeneity, transcriptome signature and functions of many tumour‐related immune cell types in the TIME have been reported in breast cancer,^18^ colorectal cancer^19^ and other cancers. Recent studies in ESCC using scRNA‐seq revealed the landscape of ESCC, including immune cells, epithelial cells and stromal cells, and elucidated the characteristics of immune suppressive cells, such as regulatory T cells and tumour‐associated macrophages, and the interactions between these cells.^20^, ^21^ However, no reports have examined the clinical implication and functions of TIL‐Bs in the TIME of ESCC using scRNA‐seq.

In the present study, we performed scRNA‐seq with ESCC samples including tumour tissues treated with/without neoadjuvant chemotherapy (NACT), normal tissues and LNs. Through these analyses, we investigated the functional heterogeneity of TIL‐B subtypes in ESCC.

## 2 METHODS

### 2.1 Patient specimens

We collected 23 samples from patients using protocols with approval by Kyushu University Certified Institutional Review Board Clinical Trials (approval number #2020‐788). All patients provided written informed consent prior to the study, and their records were de‐identified. Patient metadata are listed in Table S1.

### 2.2 Sample preparation and sequencing

Specimens were mechanically and enzymatically dissociated for scRNA‐seq. We used an online protocol for sample preparation (https://doi.org/10.17504/protocols.io.b2udqes6). Single‐cell sequencing was performed using the Chromium Single‐Cell 3′v3.1 Chemistry Library, Gel Beads and i7 Multiplex Kit (10x Genomics, Pleasanton, California, USA) in accordance with the manufacturer's protocol. A total of 8,000 to 10,000 cells were targeted per well. The libraries were sequenced on an Illumina NovaSeq 6000 (Illumina, San Diego, California, USA) and DNBSEQ‐G400 (MGI Tech, Shenzhen, China).

### 2.3 Data processing with scRNA‐seq

The sequence data were quantified and mapped to the human reference genome (GRCh38) using the Cell Ranger toolkit (ver. 4.0.0) by 10× Genomics. The Cell Ranger generated a count matrix data with filtering errors. The matrix data were imported using the Seurat R package (version 3.6.3). We performed quality control to exclude low quality cells with > 10% mitochondrial UMIs and > 5000 expressed genes. We filtered out doublets using the Doubletfinder tool.^22^

### 2.4 Data visualisation

We normalised data and identified 2,000 genes with highly variable expression after calculating a subset of features that exhibit high cell‐to‐cell variation in the data set. To regress the effects of cell cycle heterogeneity, we calculated cell cycle phase scores using canonical markers, assigned each cell a score and subtracted this source of heterogeneity from the data. We then integrated the data, performed dimensional reduction with principal component analysis (PCA) and removed batch‐effects using the Harmony method, which re‐arranged clusters to meet different batch‐effects iteratively in PCA dimensional space while keeping the diversity of batch‐effects among each cluster.^23^ We used ‘FindNeighbors’ to refine the edge weights between any two cells based on the shared overlap in the PCA space and performed ‘FindCluster’ to group cells together. Next, we ran non‐linear dimensional reduction with Uniform Manifold Approximation and Projection (UMAP) to visualise and explore the data sets.

### 2.5 Co‐detection by indexing multiple tissue staining

The co‐detection by indexing (CODEX) experiment was performed as previously described.^24^ Briefly, we mounted 4‐μm‐thick FFPE tissue sections onto poly‐l‐lysine‐coated coverslips, which were prepared according to the CODEX protocol.^24^ The coverslips were deparaffinised and rehydrated, and antigen retrieval was performed with heat. Tissues were stained with an antibody cocktail and imaged in accordance with the Akoya Biosciences CODEX protocols. Primary antibodies are listed in Table S2. Imaging was performed using KEYENCE BZX‐810 (Keyence, Osaka, Japan), an Akoya CODEX microfluidics instrument (INST2000; Akoya Biosciences, Menlo Park, California, USA), CODEX instrument manager software (v1.29.3.6) and CODEX processor software (v1.7). Light exposure and cycles are outlined in Table S3. We analysed the images using Fiji/ImageJ (National Institutes of Health, https://fiji.sc/, v1.53c) and the multiplex image viewer plugin (v1.5.0.8).

### 2.6 Differentially expressed genes and gene ontology analyses

Differentially expressed genes (DEGs) were analysed by comparison of tissue source types or clusters using a two‐sided Wilcoxon rank‐sum test with Bonferroni FDR collection. To investigate the biological function of each cluster or each B cell subtype, we performed gene ontology (GO) analysis using Metascape (https://metascape.org/gp/index.html#/main/step1).^25^ This analysis used genes up‐regulated in specific clusters (‘FindAllMarkers’ tool; comparing one cluster with all other clusters).

### 2.7 Functional enrichment analysis

Pre‐ranked gene set enrichment analysis (GSEA) was performed with the fgsea package (v1.14.0 R package).^26^ GSEA analysis was performed using Hallmark and MsigDB C7 signature gene sets (with msigdbr, v7.4.1 R package). Gene sets with *p* value < .05 were considered enriched functional states or procedures.

### 2.8 Analysis of functional gene sets

To evaluate the transcriptional dynamics in cell‐type subtypes, we defined several gene sets and evaluated the expression levels of gene sets. Gene sets are shown in Table S4.

### 2.9 Survival analysis and correlation analysis

Survival analysis of gene expression signatures were performed using GEPIA2.^27^ A total of 182 oesophageal cancer (ESCA) tumour samples were included in survival analysis. We used the NF‐κB‐related gene set and top genes up‐regulated with chemotherapy in antibody‐secreting cells (ASCs). The ESCA cohort was divided into high/low expression groups by median value (50% cut‐off). The genes in the NF‐κB‐related gene set are as follows: *NFKB1*, *REL*, *RELA*, *RELB*, *NFKB2*, *NFKBIA* and *NFKBIE*. The top genes up‐regulated with chemotherapy in ACSs are shown in Table S5. Survival analyses were performed with log‐rank Mantel–Cox test.

### 2.10 Patient cohort

The study cohort included ESCC patients who underwent surgical resection in the Department of Surgery and Oncology, Kyushu University Hospital between April 2008 and March 2020 using protocols with approval by Kyushu University Certified Institutional Review Board Clinical Trials (approval number #22002‐00). After T0 and R1/2 cases were excluded, a final total of 166 patients were examined in this study. The clinicopathological profiles of patients are summarised in Table S6.

### 2.11 Immunohistochemistry

Formalin‐fixed, paraffin‐embedded tissue samples of ESCC were sliced into 4 μm thick. The slides were deparaffined manually in xylene and rehydrated in ethanol. Endogenous peroxidase activity was blocked by methanol with 0.3% hydrogen peroxidase. For antigen retrieval, the slides were microwaved in Citrate Buffer pH 5.9 for 30 min. Slides were stained with monoclonal IGKC antibodies (clone KP‐53; Santa Cruz Biotechnology, California, USA; #sc‐59264) at a dilution of 1:100 at 4°C overnight. Slides were then incubated with secondary antibody (EnVision FLEX; DAKO/Agilent, Glostrup, Denmark; Mouse, K4001) for 1 h at room temperature and visualised with a 3,3′‐diaminobenzidine kit (Sigma–Aldrich, Darmstadt, Germany; #D5537‐5G). Slides were counterstained with Mayer's hematoxylin (Muto Pure Chemicals Co., Ltd, Tokyo, Japan; #30002).

### 2.12 Evaluation of immunohistochemistry staining

One sample analyst (S. N.) and one pathologist (Y. Y.) who were blinded to the clinical characteristics of the patients analysed immunohistochemical staining. We calculated IGKC expression using an immunostaining score, which reflects staining intensity and the proportion of positively stained cells.^28^ Staining intensity of IGKC was scored as follows: 0 = negative, +1 = weak, +2 = moderate and +3 = strong intensity. The proportion of positive cells was scored as follows: 0 = negative, 1 = 1–25%, 2 = 26–50%, 3 = 51–75% and 4 = 76–100%. The intensity score (0–3) was multiplied by the positive cell proportion score (0–4) to generate the total score, with a range of 0–12. Image processing and quantification were performed using an optical microscope (BZ‐X700; Keyence) and Fiji/ImageJ. Representative whole tissue images were obtained using image‐joint software (BZ‐Analyzer software; KEYENCE).

### 2.13 Statistics analysis

Data were analysed by GraphPad Prism (GraphPad Software, San Diego, California, USA; v9.2.0) or JMP PRO software (SAS Institute, Cary, North Carolina, USA; v16.0.0). Dunn's multiple comparisons test was performed for comparisons of several groups with proportions of cells expressing *IGHD* and *XBP1* and for immunostaining score with IGKC. Significance of differential enrichment with gene sets and specific genes was determined by Wilcoxon rank‐sum test. Correlations between clinicopathological characters and the IGKC score were determined by chi‐square test or Fisher's exact test (Table S6). Survival curves (recurrence‐free survival [RFS] and OS) were created by the Kaplan–Meier method for the 166 ESCC patients. The difference of survival curves was evaluated using the Gehan–Breslow–Wilcoxon test. Multivariable analyses were adjusted by sex, age, T stage, LN status and the IGKC expression. Cox‐regression analyses were performed to identify independent prognostic factors of RFS and OS. All statistical tests were two‐sided and *p* values < .05 were considered statistically significant.

## 3 RESULTS

### 3.1 A single‐cell transcriptome landscape of ESCC

**FIGURE 1:**
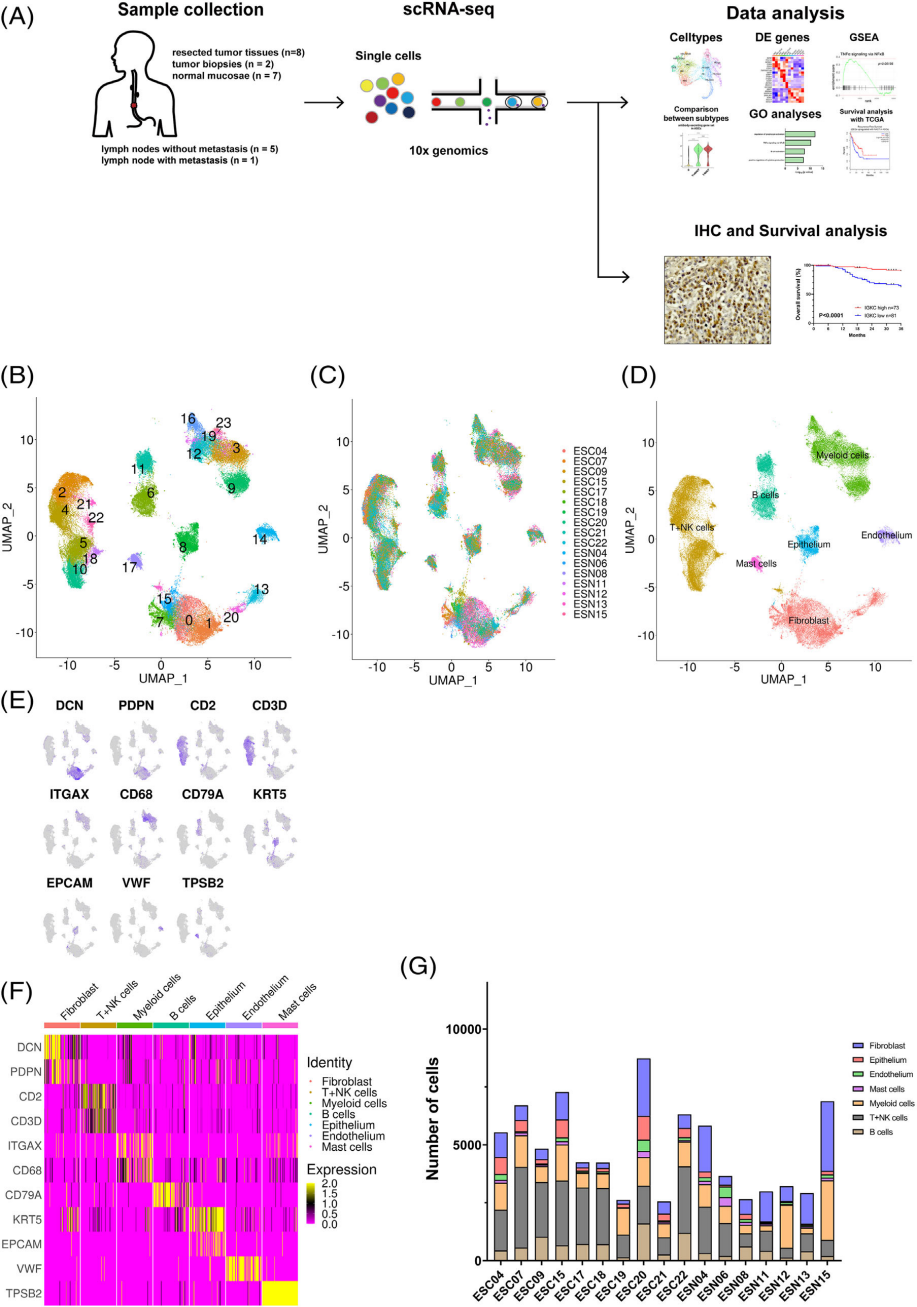
Schematics of scRNA‐seq and major cell types in ESCC. (A) Schematic illustration of the experimental workflow in our study. (B) UMAP plot showed 24 clusters from eight resected tumour tissues, two tumour biopsies and seven normal tissues. (C) The UMAP plots were coloured by sample origin. (D) UMAP plot showed seven major cell types using canonical marker genes. (E) UMAP plots showed the expression levels of canonical marker genes. (F) Heatmap of the canonical marker genes in each major cell type. (G) The number of the major cell types in the sample origins.

To examine the heterogeneity of the TIME, we performed droplet‐based scRNA‐seq in 17 samples, including 10 ESCC tumour samples (eight surgical samples and two endoscopic samples) and seven non‐tumour samples (Figure 1A and Table S1). After quality control and removing 2,899 doublets, 81,246 cells remained. Following gene expression normalisation and PCA, we performed graph‐based clustering on the PCA, applied batch correction (using the Harmony method^23^) and visualised via UMAP plots. We grouped the cells into 24 clusters (Figure 1B). UMAP plots showed that various cells were consisted of each sample origin, tissue histology (normal or tumour) and treatment with/without NACT (Figures 1C and S1A, B). Using canonical lineage marker genes, the clusters were annotated to seven known cell‐types: fibroblasts (*DCN* and *PDPN*; 16,823 cells), T+NK cells (*CD2* and *CD3D*; 28,248 cells), myeloid cells (*ITGAX* and *CD68*; 17,114 cells), B cells (*CD79A*; 9,673 cells), epithelium (*KRT5* and *EPCAM*; 5,160 cells), endothelium (*VWF*; 2,389 cells) and mast cells (*TPSB2*; 1,839 cells) (Figures 1D–F). UMAP plots classified with such cell types were showed by the tissue source type (normal samples (N), tumour samples without NACT (T‐nNACT) and tumour samples with NACT (T‐NACT)) (Figure S1C). The number of each cell type was different among their sample origins (Figure 1G).

### 3.2 Classification of TIL‐B subtypes in ESCC

To investigate changes in the subtypes and functions of TIL‐Bs during chemotherapy in ESCC, we analysed TIL‐B subtypes. First, we extracted B cell clusters based on the expressions of major markers and re‐clustered. We identified new B cell clusters in this data set. UMAP plot showed that these clusters were shared across sample origins, tissue histology and treatments (Figures S2A–C). Using known genes, we identified five B cell major subtypes, including naive B cells (NBCs; *IGHD* and *TCL1A*), activated B cells (ABCs; *CD69*, *CD83* and *EGR1*), which is antigen‐specific activated B cell subtype and committed to memory B cell (MBC),^29^ MBCs (*TNFRSF13B*, *CD27*, *CRIP1* and *ITGB1*), germinal centre B cells (GCBs; *AICDA*, *RGS13* and *GCSAM*) and ASCs (*XBP1* and *PRDM1*) (Figures S2D and E). ASC classifications were determined by their heavy chains (*IGHA1*, *IGHD*, *IGHE*, *IGHG1* and *IGHM*) (Figure S2F). During NBC differentiation into ASCs, the expression levels of major histocompatibility complex (MHC) class I/II changed; ASCs completely lost the expression of MHC class II and lost antigen‐presenting function (Figure S2G).

**FIGURE 2:**
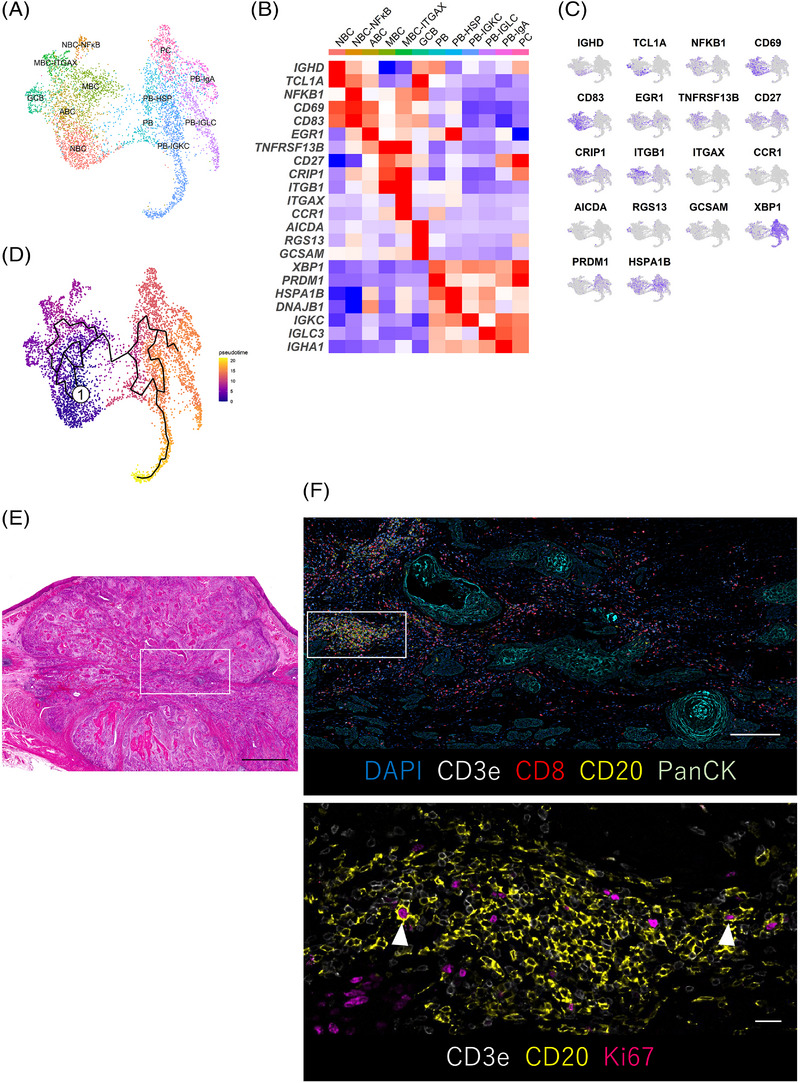
The subtypes of TIL‐Bs in ESCC. (A) UMAP plot was color‐coded by 12 subtypes of TIL‐Bs on the basis of representative genes. (B) Heatmap indicated representative genes of B cells in each subtype. (C) UMAP plots showed the expression levels of representative genes in TIL‐Bs. (D) Trajectory analysis was performed between B cell subtypes. (E and F) CODEX multiple staining images showed intratumoural germinal centre B cells (CD20^+^, Ki67^+^; white arrowheads). Representative histologic section of ESCC (H&E, merged images using image‐joint software) (E). The CODEX multiple staining images of DAPI, CD3e, CD8, CD20 and PanCK (F, top) and high magnification images of CD3e, CD20 and Ki67 (F, bottom). Regions of interest were shown in the white box. Scale bars, 2000 μm (E), 1000 μm (F, top) and 100 μm (F, bottom).

For a more detailed assessment, B cells were further classified into 12 subtypes (Figures 2A–C). Among NBCs, we identified the NBC–NF‐κB subtype that highly expressed the NF‐κB pathway transcripts. We also identified the MBC–ITGAX subtype, which expressed the canonical genes of MBCs and characteristic genes, such as *ITGAX* and *CCR1*. To elucidate the molecular characteristics of the MBC–ITGAX subtype, we performed GSEA. The MBC–ITGAX subtype was enriched for signalling pathways such as B cell Fluarix up, which meant acquiring adaptive immunity in B cells after exposure to Fluarix (influenza virus vaccine) and transforming growth factor‐β (TGFβ) (Figure S2H). About ASCs, a previous study reported that expression of BLIMP1 (encoded by *PRDM1*) identified the ASC states, such as plasmablasts (PBs) and plasma cells (PCs), and PBs display a lower level of BLIMP1 expression than PCs.^30^ We found two subtypes (PBs and PCs) based on differences in the expression levels of *PRDM1* in ASCs. Additionally, among PBs, we identified a subtype expressing heat shock protein (HSP)‐related genes (PB‐HSP) and three subtypes by specific expression of immunoglobulin subtypes (PB‐IGKC, PB‐IGLC and PB‐IgA). We next performed the trajectory analysis to investigate the potential transition between B cell subtypes using the Monocle analysis toolkit.^31^ The pseudotime trajectory analysis showed that NBCs transdifferentiate into ABCs then into MBCs (Figure 2D). On the other hand, we found that GCBs existed in tumour sites using scRNA‐seq. GCBs, T cells and B cells construct the tertiary lymphoid structure (TLS), which is an ectopic lymphoid tissue and existed nearby or inside tumour. TLS is known to stimulate the adaptive anti‐tumour immune response.^32^ Therefore, we performed CODEX,^24^ with resected human ESCC tissues to evaluate the GCB existence. We found aggregated structures of T cells and B cells, indicating TLS, and GCBs (CD20^+^Ki67^+^) within B cell aggregates (Figures 2E,F and S2I and Tables S2 and S3). The present scRNA‐seq data showed that there were novel and unique TIL‐B subtypes in the ESCC microenvironment.

### 3.3 Functional characterisation of TIL‐B subtypes

We then investigated B cell‐related functions, such as B cell‐secreting molecules (interleukins, cytokines and chemokines), the B cell receptor (BCR) signalling, MYC pathway and PD‐1 signaling^33^, ^34^ in each TIL‐B subtype (Figure S3A). The expressions of these B cell function‐related genes were down‐regulated in ASCs. *CCL17* and *CCL22* encode proteins that are secreted by B cells with CD40 signalling and attract follicular helper T cells (Tfh cells) via engaging the chemokine receptor CCR4 in Tfh cells.^35^ We found that *CCL17* and *CCL22* were highly expressed in the MBC–ITGAX subtypes. We also found up‐regulation of the MYC pathway genes and PD‐1 signalling genes in the MBC–ITGAX subtype.

**FIGURE 3:**
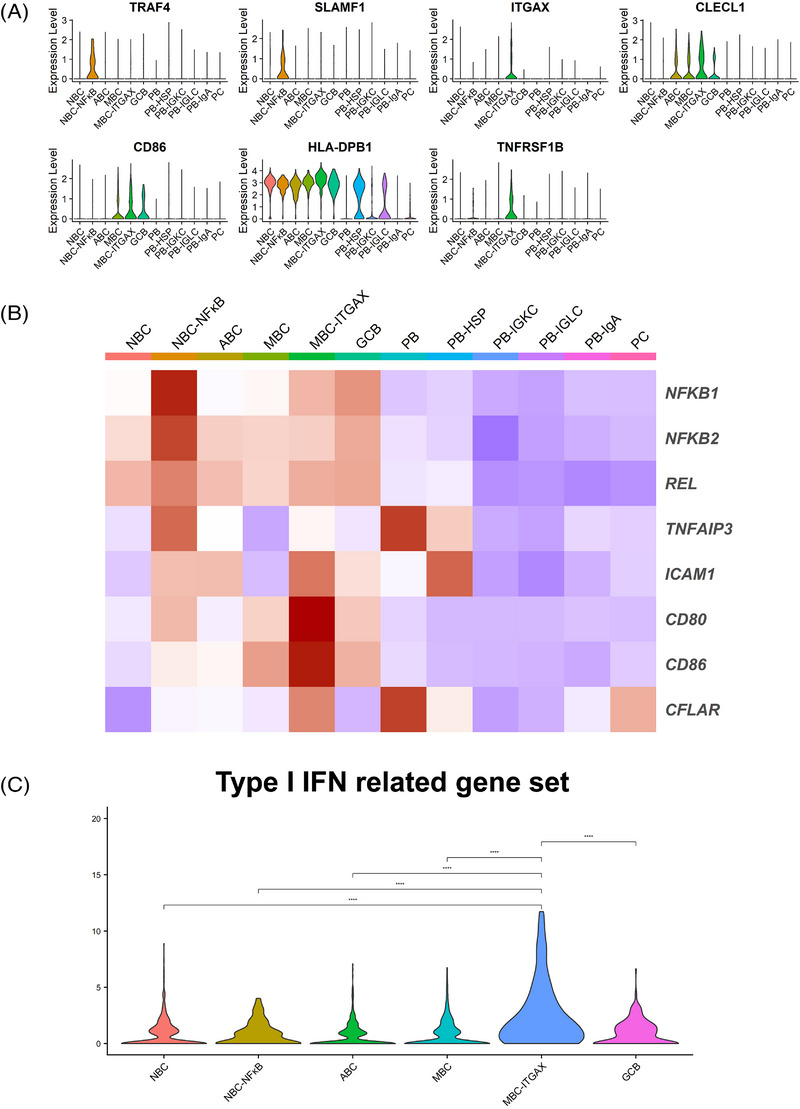
The expression of specific functional genes in TIL‐B subtypes. (A) Violin plots showed the expression of specific functional genes in each subtype. (B) Heatmap indicated the expression of functional genes in each subtype. (C) Violin plots showed the expression of the type I IFN‐related gene set in B cells except for ASCs. Significance of expression (*p* value) between tissue source types was determined by Wilcoxon rank‐sum test, with all *p* values adjusted using Bonferroni correction. ^****^ *p* < .0001

We next analysed the unique functions by the specific genes expressed in each subtype. The PB‐HSP subtype expressed HSP‐related genes (such as *DNAJA1*, *DNAJB1*, *HSPE1* and *HSPA8*) most highly among ASC subtypes (Figure S3B). Notably, the NBC–NF‐κB subtype expressed genes that activate the NF‐κB pathway that is associated with B cell maturation and survival (*TRAF4, NFKB1*, *NFKB2*, *REL* and *TNFAIP3*)^36^, ^37^ (Figures 3A and B). Among the TIL‐B subtypes, the NBC–NF‐κB subtype expressed the highest levels of *SLAMF1*. *SLAMF1* encodes a protein that maintain interactions with Tfh cells to form the germinal centre.^38^ The MBC–ITGAX subtype highly expressed *ITGAX*, *CLECL1* and *CD86* (Figures 3A and B). These genes are associated with cell adhesion and co‐stimulation for antigen presentation.^39^, ^40^ This subtype also expressed *HLA‐DPB1* and CD40 signalling‐related genes (*ICAM1*, *CD80*, *CD86* and *CFLAR*), which are essential for antigen presentation to T cells. *TNFRSF1B* encodes TNFR2, which triggers the NF‐κB pathway possibly to activate MBCs.^37^ This gene was up‐regulated in the MBC–ITGAX subtype. Type I IFN induces the up‐regulation of surface molecules on B cells, such as CD86 and MHC class II.^41^ The MBC–ITGAX subtype expressed the highest level of the type I IFN‐related gene set among TIL‐B subtypes (Figure 3C and Table S4).

### 3.4 Differences in proportions and functions of TIL‐Bs by the tissue source type

**FIGURE 4:**
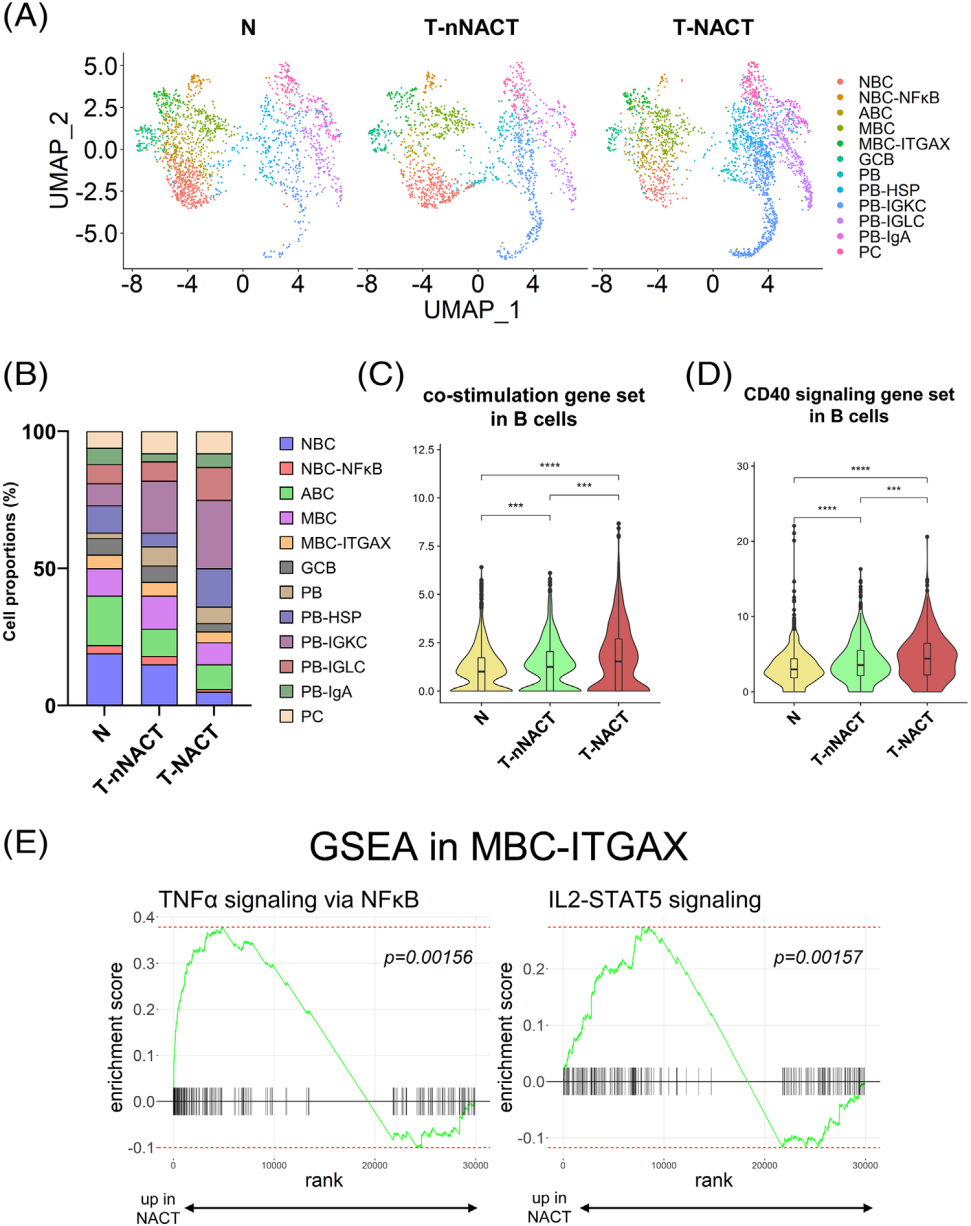
Changes in proportions and functions of TIL‐B subtypes by the tissue source type. (A) UMAP plots showed 12 subtypes of TIL‐Bs by the tissue source type. (B) Bar plots showed the proportions of 12 subtypes of TIL‐Bs by the tissue source type. (C and D) Violin plots showed the expressions of co‐stimulation (C) and CD40 signalling‐related gene set (D) in B cells except for ASCs by the tissue source type. Significance of these gene set enrichment (*p* value) between tissue source types was determined by Wilcoxon rank‐sum test. Boxplots included centreline, median; box limits, upper and lower quartiles; whiskers at most 1.5× the interquartile range past upper and lower quartiles. ^***^ *p* < .001, ^****^ *p* < .0001. (E) GSEA results showed the TNFα signalling via NF‐κB pathway and IL2‐STAT5 signalling pathway was enriched in the MBC–ITGAX subtype from NACT patients compared with nNACT patients.

To investigate the changes of TIL‐Bs during chemotherapy, we plotted clustering with the TIL‐B subtypes (Figure 4A) and investigated the proportions of subtypes by the tissue source type (Figure 4B). The plots showed that the proportion of NBCs (including NBC and NBC–NF‐κB) in tumour tissues with NACT was less than that in tumour tissues without NACT, which was less than that in normal tissues. Additionally, the proportion of ASCs (including PB, PB‐HSP, PB‐IGKC, PB‐IGLC, PB‐IgA and PC) was larger in tumour tissues with NACT than in tumour tissues without NACT, which was more than in normal tissues. We focused on functional changes associated with NACT in TIL‐Bs. B cells function as professional antigen‐presenting cells and express several co‐stimulatory molecules, such as CD40.^42^ The co‐stimulation and CD40 signalling‐related gene sets were up‐regulated in B cells except for ASCs with NACT (Figures 4C and D). We next investigated the functional changes in the MBC–ITGAX subtype because the present data revealed that this subtype highly expressed genes associated with the co‐stimulation gene set. In GSEA using DEGs associated with NACT, we found that the MBC–ITGAX subtype showed enrichment of tumour necrosis factor‐α (TNFα) signalling via NF‐κB pathway and IL2‐STAT5 signalling pathway (Figure 4E), suggesting that NACT enhanced cellular activation in the MBC–ITGAX subtype.^43^

### 3.5 Changes in the proportion and functions of NBCs during NACT

**FIGURE 5:**
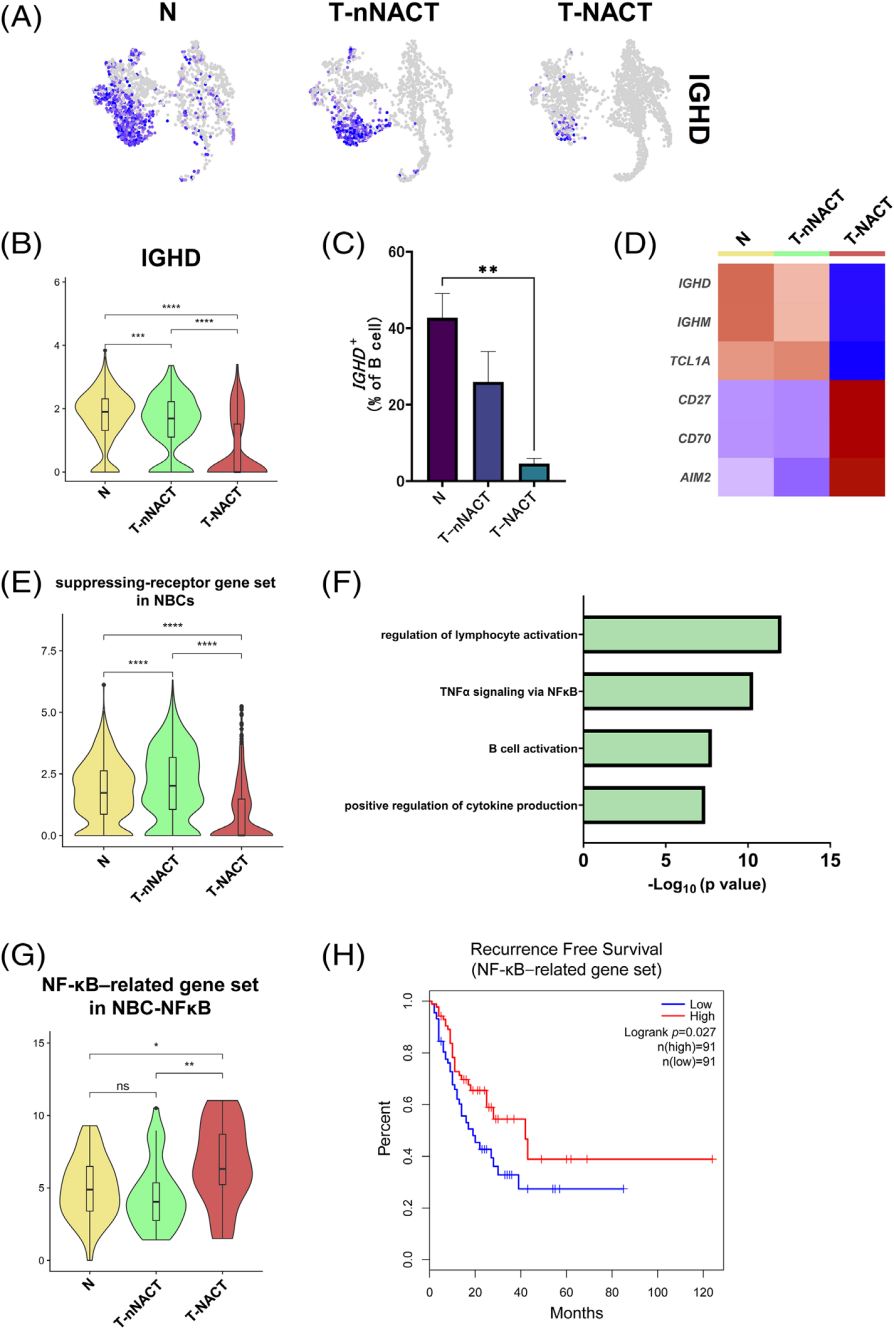
Decrease of the proportion and functional changes in NBCs during chemotherapy. (A) UMAP plots showed expression levels of *IGHD*, which is a canonical marker gene for naive B cells (NBCs), by the tissue source type. (B) Violin plots showed the expression of *IGHD* in NBCs by the tissue source type. Significance of *IGHD* expression (*p* value) between tissue source types was determined by Wilcoxon rank‐sum test. Boxplots included centreline, median; box limits, upper and lower quartiles; whiskers at most 1.5× the interquartile range past upper and lower quartiles. ^***^ *p* < .001, ^****^ *p* < .0001. (C) Bar plots showed the percentage of *IGHD* ^+^ cells to total B cells by the tissue source type (N; *n* = 7, T‐nNACT; *n* = 5, T‐NACT; *n* = 5). Mean + SEM shown. Dunn's multiple comparisons test was performed. ^**^ *p* < .01. (D) Heatmap showed the expression of DEGs among tissue source types in NBCs. (E) Violin plots showed the expression of the suppressing‐receptor gene set in NBCs. Significance of this gene set enrichment (*p* value) between tissue source types was determined by Wilcoxon rank‐sum test. Boxplots included centreline, median; box limits, upper and lower quartiles; whiskers at most 1.5× the interquartile range past upper and lower quartiles. ^****^ *p* < .0001. (F) The enriched Gene Ontology term for the top 100 signature genes in the NBC subtype with T‐NACT using Metascape. (G) Violin plots showed the expressions of the NF‐κB‐related gene set by the tissue source type. Significance of this gene set enrichment (*p* value) between tissue source types was determined by Wilcoxon rank‐sum test. Boxplots included centreline, median; box limits, upper and lower quartiles; whiskers at most 1.5× the interquartile range past upper and lower quartiles. ns; not significant, ^*^ *p* < .05, ^**^ *p* < .01. (H) Survival analysis based on the NF‐κB‐related gene set in TCGA‐ESCA (*n* = 182). Log‐rank Mantel Cox–test.

We focused on NBCs during NACT. The proportion of NBCs was the lowest in tumour tissues with NACT among the three tissue types (Figure 4B). The expression level of *IGHD*, which is major marker of NBCs, and the proportion of *IGHD*‐positive cells was the lowest in tumour cells with NACT among the three tissue source types (Figures 5A–C). We next examined the differences in function‐related gene expression with NBCs compared with each tissue source type and found that B cell activation markers (*CD27*, *CD70* and *AIM2*) were strongly up‐regulated in NBCs with NACT (Figure 5D). B cells express suppressing‐receptors to inhibit BCR signalling.^44^ We found that the suppressing‐receptor gene set was down‐regulated in NBCs with NACT (Figure 5E). GO enrichment analysis showed that DEGs in the NBC subtype with NACT compared with the subtype without NACT were enriched for regulation of lymphocyte activation, TNFα signalling via NF‐κB, B cell activation and positive regulation of the cytokine production pathway (Figure 5F). We next focused on the NF‐κB pathway, which is involved in B cell maturation and adaptive immunity.^45^ The NF‐κB‐related gene set was up‐regulated in the NBC–NF‐κB subtype with NACT (Figure 5G). To investigate the clinical implication of this pathway activation in oesophageal cancer, we performed survival analysis using TCGA data. We found significant correlations between prolonged RFS and the NF‐κB‐related gene set (*p* = .027) (Figure 5H).

### 3.6 Changes in the proportion and functions of ASCs during NACT

**FIGURE 6:**
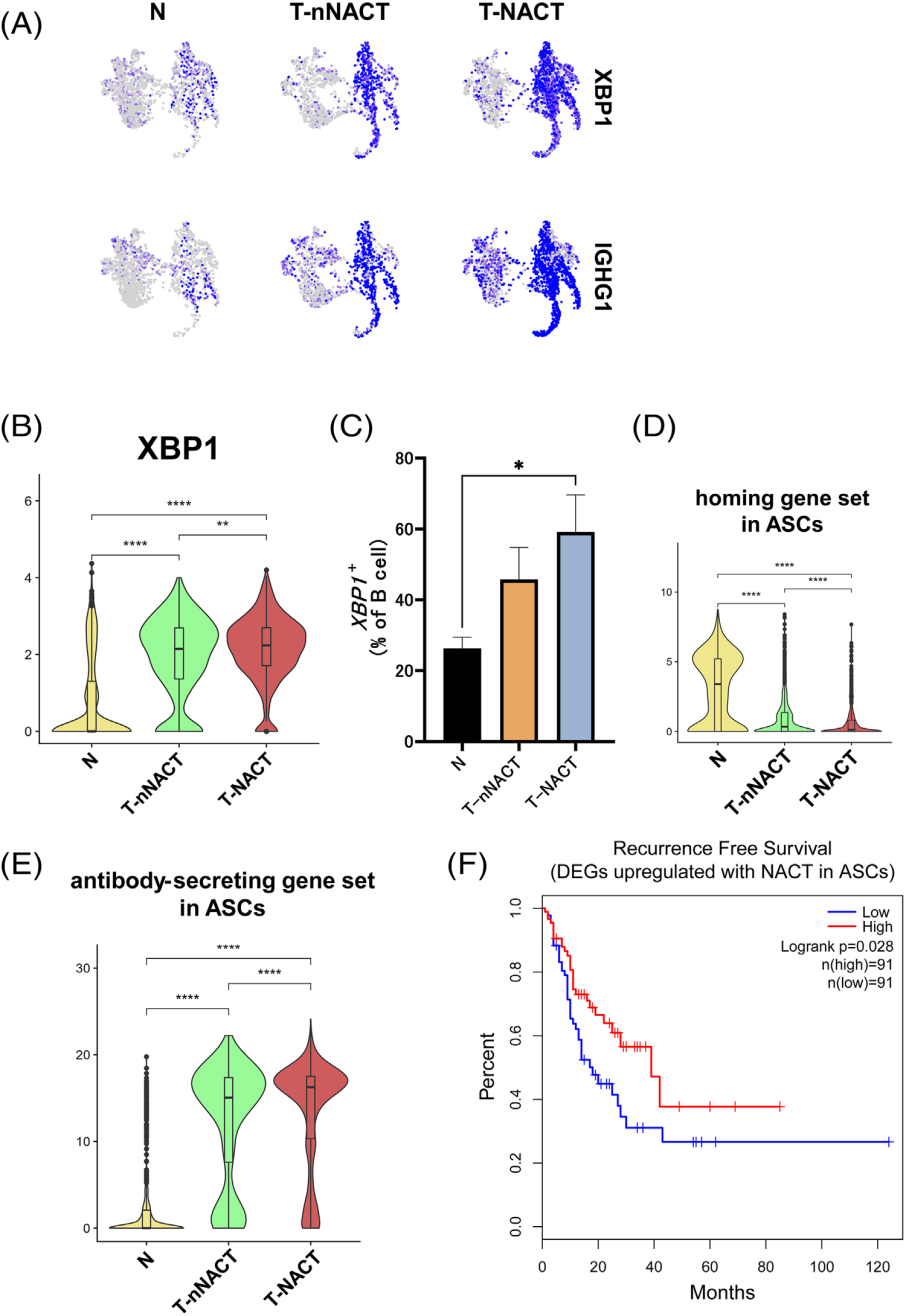
Increase of the proportion and enhancement of functions in ASCs during chemotherapy. (A) UMAP plots showed the expression levels of *XBP1* and *IGHG1*, which are the canonical marker genes for antibody‐secreting cells (ASCs), by the tissue source type. B) Violin plots showed the expression of *XBP1* in ASCs by the tissue source type. Significance of *XBP1* expression (p‐value) between tissue source type was determined by Wilcoxon rank‐sum test. Boxplots included centreline, median; box limits, upper and lower quartiles; whiskers at most 1.5× the interquartile range past upper and lower quartiles. ^**^ *p* < .01, ^****^ *p* < .0001. (C) Bar plots showed the percentage of *XBP1* ^+^ cells in total B cells by the tissue source type (N; *n* = 7, T‐nNACT; n = 5, T‐NACT; *n* = 5). Mean + SEM shown. Dunn's multiple comparisons test was performed. ^*^ *p* < .05. (D) Violin plots showed the expressions of the homing gene set in ASCs by the tissue source type. Significance of this gene set enrichment (*p* value) between tissue source types was determined by Wilcoxon rank‐sum test. Boxplots included centreline, median; box limits, upper and lower quartiles; whiskers at most 1.5× the interquartile range past upper and lower quartiles. ^****^ *p* < .0001. (E) Violin plots showed the expressions of the antibody‐secreting gene set in ASCs by the tissue source type. Significance of this gene set enrichment (*p* value) between tissue source types was determined by Wilcoxon rank‐sum test. Boxplots included centreline, median; box limits, upper and lower quartiles; whiskers at most 1.5× the interquartile range past upper and lower quartiles. ^****^ *p* < .0001. (F) Survival analysis based on DEGs up‐regulated with NACT in ASCs using TCGA‐ESCA data (*n* = 182). Log‐rank Mantel Cox–test.

We then focused on the ASCs during NACT. The proportion of ASCs was the highest in tumour tissues with NACT among the three tissue types (Figure 4B). ASCs in tumour tissues with NACT expressed the highest levels of *XBP1* and *IGHG1* among the three tissue source types (Figures 6A and B) and the proportion of *XBP1*‐positive cells was the largest in tumour tissues with NACT among the three tissue source types (Figure 6C). In general, mature ASCs migrate from generated sites to survival niches, especially bone marrow. We evaluated the homing gene set including ASC receptors related with homing. This gene set was significantly down‐regulated in ASCs in tumour tissues with NACT compared with clusters in normal tissues (Figure 6D). Additionally, to evaluate ASC abilities to secrete antibodies after class switching, we analysed the expression of the antibody‐secreting gene set. We found that the gene set was strongly enriched in ASCs treated with NACT (Figure 6E). To evaluate the prognostic impact of functional changes in ASCs during NACT on oesophageal cancer, we examined the relationship between DEGs associated with NACT and outcomes of oesophageal cancer using TCGA data. NACT‐induced DEGs in ASCs (Table S5) were significantly correlated with RFS in oesophageal cancer (*p* = .028) (Figure 6F).

### 3.7 A single‐cell landscape of LNs

In LNs, many immune cells carry out active immune responses to TAAs. To investigate the functional heterogeneity of B cells in a metastatic LN, we analysed changes in subtypes and functions of B cells derived from LNs using scRNA‐seq. We obtained six resected samples from three normal LNs treated with only surgery (nM‐nNACT), two normal LNs treated with NACT and surgery (nM‐NACT) and one metastatic LN treated with NACT and surgery (M‐NACT). After quality control and removing 2,899 doublets, 35,749 cells remained. Following performing PCA, applying batch correction, single‐cells were visualised in the UMAP plot and classified 16 cell type clusters (Figure S4A). UMAP plots showed that these cells from LNs were shared across sample origins, tissue histology (without metastasis (nMetastasis) or with metastasis) and treatments (Figures S4B–D). On the basis of the expression of canonical marker genes and characteristic genes, we identified three major cell types (T+NK cells, B cells and myeloid cells) (Figures S4E–G). Additionally, to assess the contamination of tumour cells in metastatic samples, we examined the expression of oesophageal epithelium genes (*KRT5, KRT19*, *EPCAM* and *SOX4*) and found almost no cells that expressed these genes (Figure S4H). The number of major cell types is shown in each sample origin (Figure S4I). These data indicated that LNs were mainly composed of T+NK cells and B cells.

### 3.8 Profile of B cell subtypes in LNs, and characteristics and variations

**FIGURE 7:**
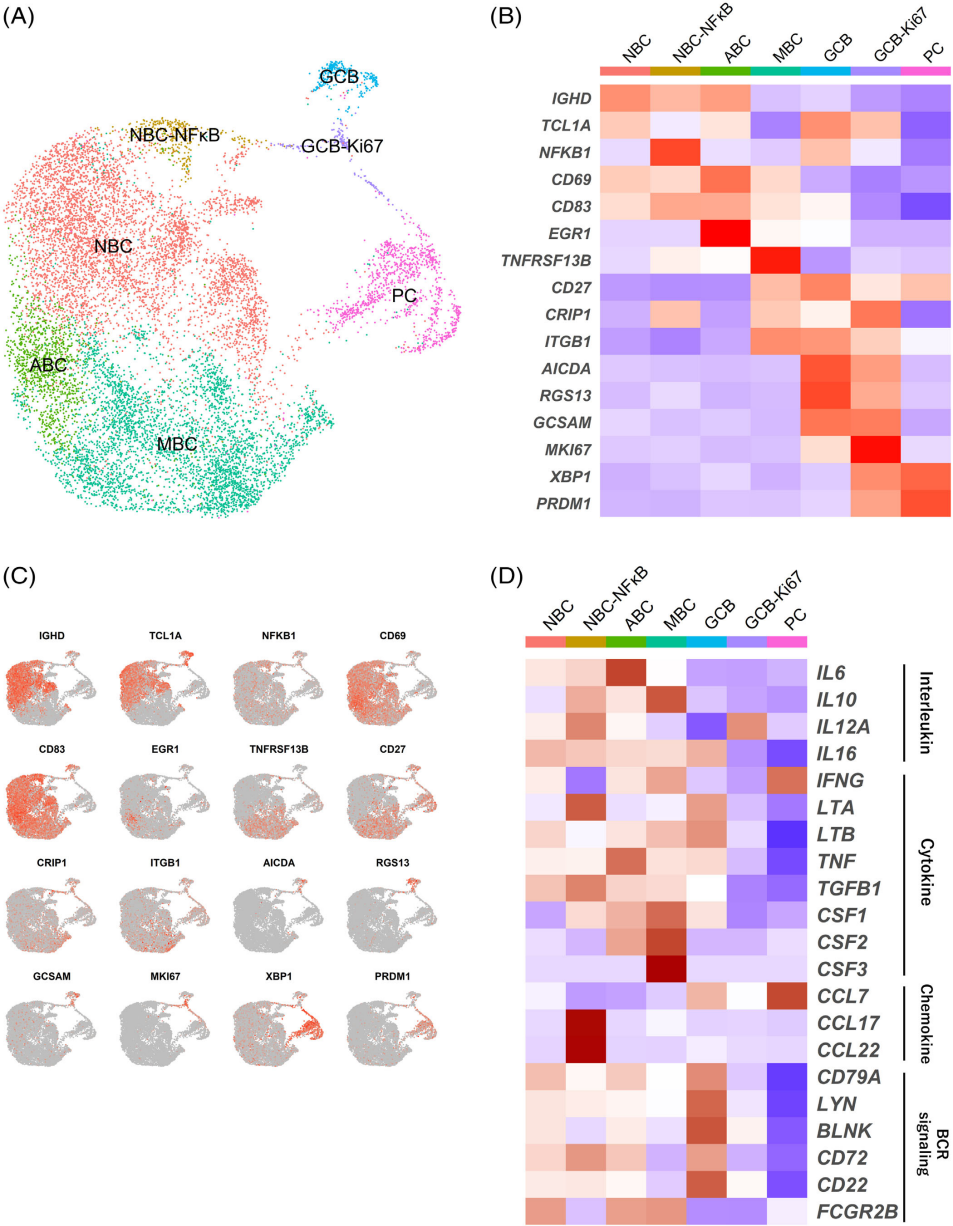
The subtypes of B cells in LNs. (A) UMAP plot was color‐coded by seven subtypes of B cells in LNs on the basis of representative genes. (B) Heatmap showed representative genes of B cells in each subtype. (C) UMAP plots showed the expressions of representative genes. (D) Heatmap showed the expression of B cell function‐associated genes in each subtype.

We next analysed the heterogeneity of B cells in LNs. We re‐clustered B cells in LNs and identified B cell clusters across all patients, tissue histology and treatments (Figures S5A–C). On the basis of their unique gene expressions, we classified B cells in LNs into seven detailed subtypes (Figures 7A–C). The GCB‐Ki67 subtype expressed *MKI67* (which encodes Ki67) at a higher level than the GCB subtype. The expression levels of B cell function‐related genes in ASCs were lower than in other B cells same as the primary tumour (Figure 7D). In the NBC–NF‐κB subtype, *CCL17* and *CCL22* were most highly expressed among B cell subtypes in LNs. To characterise the GCB‐Ki67 subtype, we evaluated DEGs in the GCB‐Ki67 subtype compared with the GCB subtype. The GCB‐Ki67 subtype showed up‐regulation of IgG‐Memory/PC down, which meant that the differentiated state of GCB‐Ki67 was more similar to that of ASCs than to MBCs, Naive/GC B cell down, which indicated that the differentiated state of the GCB‐Ki67 subtype was more analogous to that of GCBs than to NBCs, and the E2F pathway, indicating cell proliferation (Figure S5D).

**FIGURE 8:**
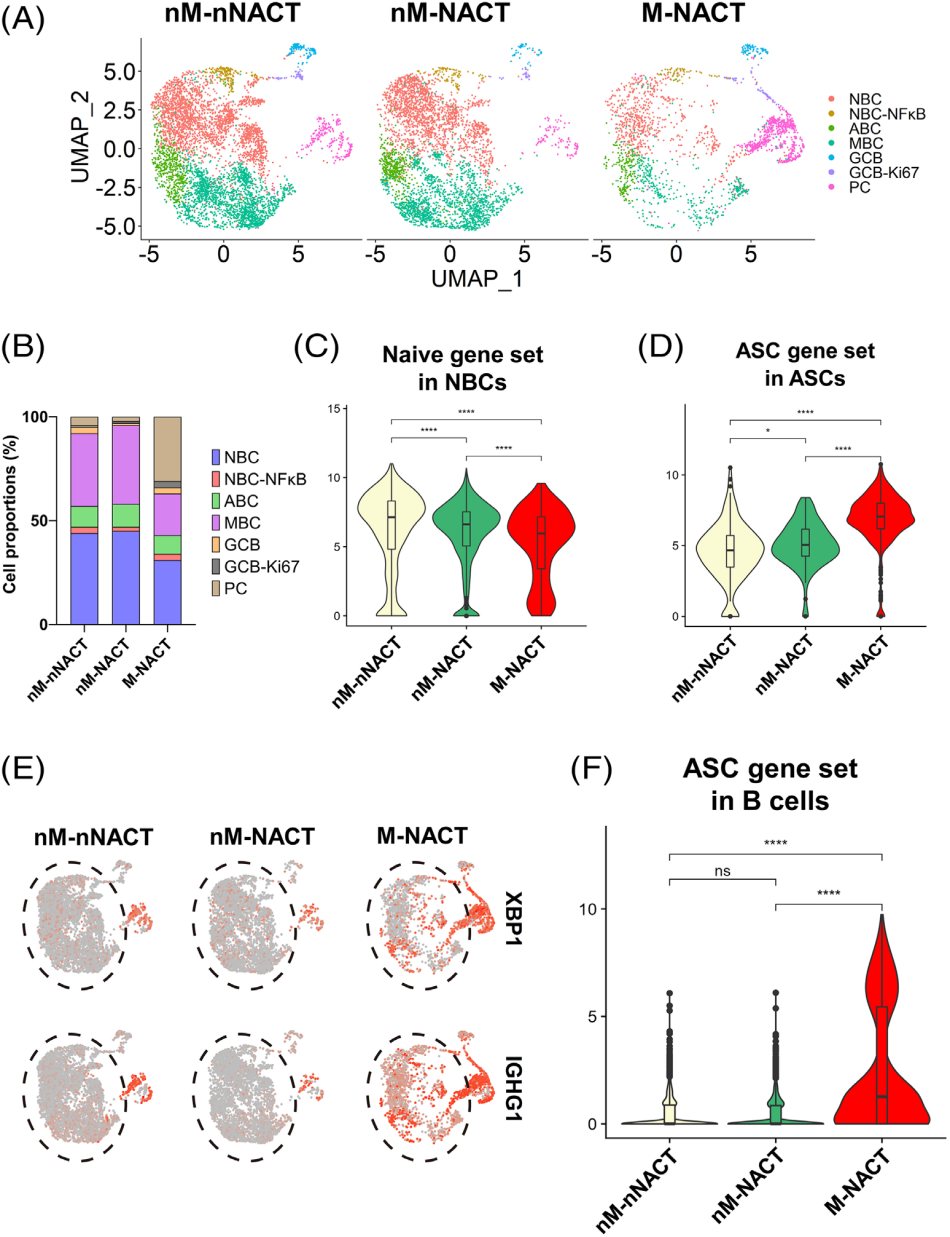
Differentiation of B cells in a metastatic LN. (A) UMAP plots showed seven subtypes of B cells in LNs by the tissue source type. (B) Bar plots showed the proportions of seven B cell subtypes in LNs in the tissue source types. (C and D) Violin plots showed the expression of Naive gene set in NBCs (C) and ASC gene set in ASCs (D). Significance of these gene set enrichment (*p* value) between tissue source types was determined by Wilcoxon rank‐sum test. Boxplots included centreline, median; box limits, upper and lower quartiles; whiskers at most 1.5× the interquartile range past upper and lower quartiles. ^*^ *p* < .05, ^****^ *p* < .0001. (E) UMAP plots showed expression levels of *XBP1* and *IGHG1* by the tissue source type. Black dotted frames showed B cells except for ASCs. (F) Violin plots showed the expression of the ASC gene set in B cells except for ASCs. Significance of this gene set enrichment (*p* value) between tissue source types was determined by Wilcoxon rank‐sum test. Boxplots included centreline, median; box limits, upper and lower quartiles; whiskers at most 1.5× the interquartile range past upper and lower quartiles. ns; not significant, ^****^ *p* < .0001.

In drainage LNs of primary tumours, the proportion of B cells increased and systemic humoral immune response to tumours were activated.^46^ To elucidate the characteristics of B cell subtypes in each tissue source type of LNs, we investigated differences in several B cell subtypes among the tissue source types. UMAP plot exhibited the distribution of B cell subtypes in each tissue source type and their relative proportions are shown across the tissue source types (Figures 8A and B). The ASC proportion was the largest in a metastatic LN treated with NACT among these tissue source types and this result was similarity to ASCs in the TIME. We scored the Naive gene set in NBCs and the ASC gene set in ASCs. Similar to results in the analyses of the TIME in primary tumours, NBCs in a metastatic LN showed the lowest expression levels in the Naive gene set among the three tissue source types (Figure 8C). By contrast, ASCs in a metastatic LN showed the most enriched ASC gene set among the tissue source types (Figure 8D). Moreover, B cell subtypes except for ASCs in a metastatic LN expressed the highest levels of *XBP1* and the ASC gene set in these tissue source types (Figures 8E and F). We also evaluated chemotherapy‐induced changes in LNs. B cells in LNs with NACT showed increased expression levels of *TNF* (which encodes TNFα) and *HLA‐DQA2* (which encodes MHC class II) compared with expressions in LNs without NACT (Figures S6A and B).

### 3.9 The relationship between ASCs in the TIME and prognosis in ESCC cases

**FIGURE 9:**
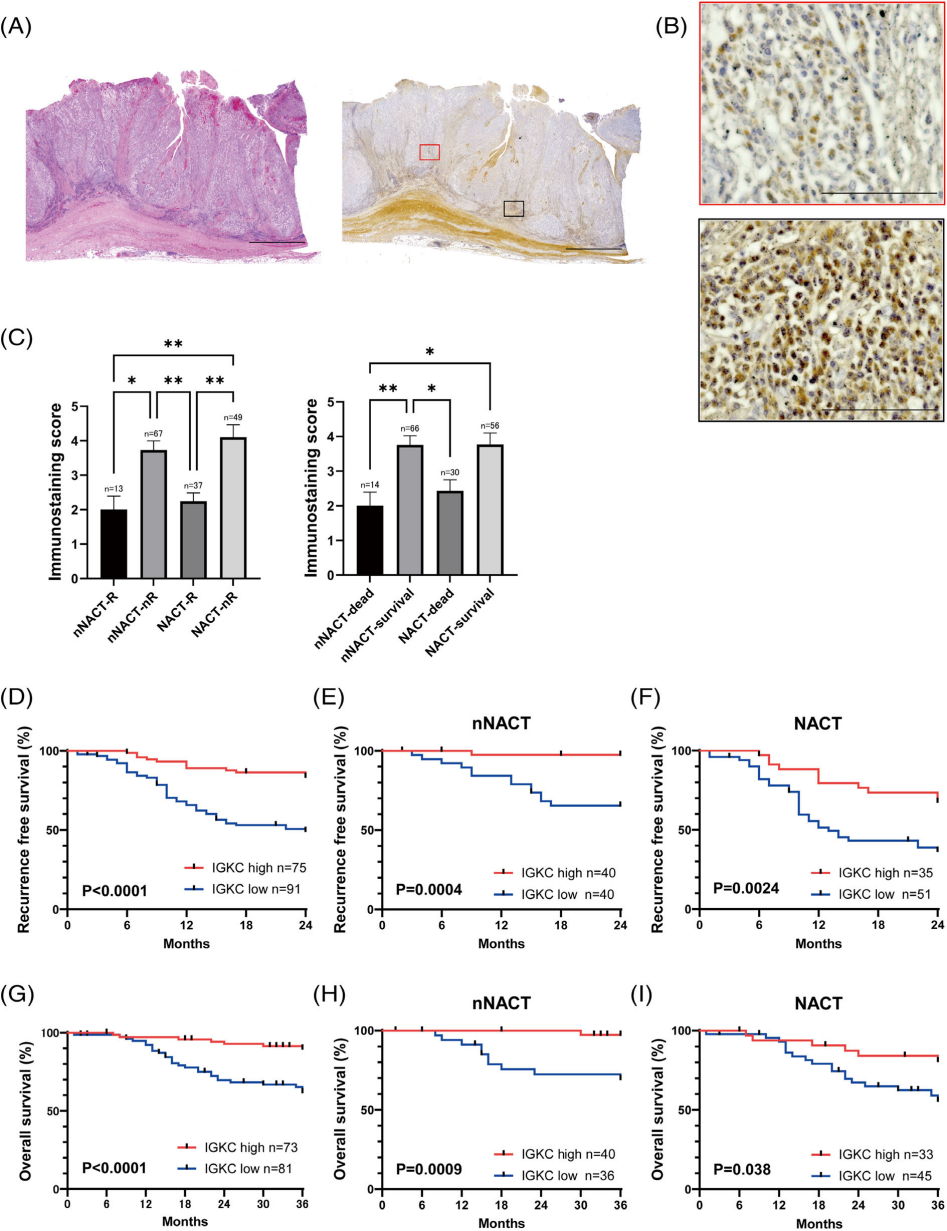
The relationship between ASCs in the TIME and prognosis in ESCC patients. (A) Representative images of H&E staining (left) and IGKC immunostaining (right), which were merged using image‐joint software. Scale bars, 3500 μm. (B) Representative images of IGKC immunostaining intratumour (×200, top, red) and peritumour (×200, bottom, black). Scale bars, 100 μm. (C) Expression of IGKC in 166 ESCC patients, about recurrence cases (left) and survival cases (right). Mean + SEM shown. Dunn's multiple comparisons test was performed. ^*^ *p* < .05, ^**^ *p* < .01. (D–F) Kaplan–Meier curves for recurrence‐free survival with IGKC high or low expression in 166 ESCC patients (D), including 80 ESCC patients without NACT (E) and 86 ESCC patients with NACT (F). *p* Value was determined with Gehan–Breslow–Wilcoxon test. (G–I) Kaplan–Meier curves for overall survival with IGKC high expression or low expression in 154 ESCC patients (G), including 76 ESCC patients without NACT (H) and 78 ESCC patients with NACT (I). P‐value was determined with Gehan–Breslow–Wilcoxon test.

To investigate the localisation of ASCs in the ESCC TIME and its correlation with prognosis, we evaluated the expression of immunoglobulin κ C (IGKC) in ESCC samples using immunohistochemistry (IHC). In previous IHC analyses, IGKC was reported to be highly expressed in ASCs and might be preferable to detect ASCs.^47^ IGKC staining intensity was classified into four scores (0∼+3) (Figure S7). We more frequently observed ASCs in peritumoural regions than in intratumoural regions (Figures 9A and B). Furthermore, in our cohort (Table S6), the IGKC expression score was remarkably high in recurrence‐free and survival patients (Figure 9C). In addition, we found that favourable 2‐year RFS and 3‐year OS were associated with the high levels of IGKC expression (Figure 9D–I). Multivariate survival analysis indicated that late stages at T3/T4, the presence of LN metastasis and IGKC expression were independent prognosis factors of RFS (hazard ratio (HR) = 2.0, 95% confidence interval (CI): 1.2–3.5, *p* = .013; HR = 2.5, 95% CI: 1.3–4.8, *p* = .0058; HR = 0.23, 95% CI: 0.12–0.45, *p* < .0001, respectively) and OS (HR = 2.3, 95% CI: 1.1–4.7, *p* = .021; HR = 2.3, 95% CI: 1.02–5.2, *p* = .049; HR = 0.20, 95% CI: 0.086–0.47, *p* = .0002, respectively) in ESCC patients (Tables 1 and 2). These data suggest that ASCs in the TIME were correlated with a favourable prognosis in ESCC patients.

**TABLE 1:** Univariate and multivariate analyses of RFS and OS with IGKC expression and clinicopathological characteristics in ESCC patients

			Univariate			Multivariate		
			HR	95% CI	*p* Value^a^	HR	95% CI	*p* Value^a^
**RFS**								
	Sex							
		Female	1 (Ref)			1 (Ref)		
		Male	1.1	0.51‐2.5	.76	1.1	0.50‐2.5	.78
	Age							
		<65^b^	1 (Ref)			1 (Ref)		
		≥65	1.1	0.66‐1.9	.70	1.4	0.82‐2.4	.22
	T stage^c^							
		T1, 2	1 (Ref)			1 (Ref)		
		T3, 4	**2.5**	**1.5‐4.2**	**.0007**	**2.0**	**1.2‐3.5**	**.013**
	Lymph node metastasis							
		Negative	1 (Ref)			1 (Ref)		
		Positive	**3.2**	**1.7‐5.9**	**.0003**	**2.5**	**1.3‐4.8**	**.0058**
	IGKC expression							
		Low	1 (Ref)			1 (Ref)		
		High	**0.25**	**0.13‐0.48**	**<.0001**	**0.23**	**0.12‐0.45**	**<.0001**
**OS**								
	Sex							
		Female	1 (Ref)			1 (Ref)		
		Male	1.1	0.38‐3.0	.90	0.93	0.32‐2.7	.89
	Age							
		<65^b^	1 (Ref)			1 (Ref)		
		≥65	1.2	0.63‐2.4	.55	1.7	0.88‐3.5	.11
	T stage^c^							
		T1, 2	1 (Ref)			1 (Ref)		
		T3, 4	**2.6**	**1.4‐5.1**	**.0044**	**2.3**	**1.1‐4.7**	**.021**
	Lymph node metastasis							
		Negative	1 (Ref)			1 (Ref)		
		Positive	**3.1**	**1.4‐6.8**	**.0052**	**2.3**	**1.02‐5.2**	**.049**
	IGKC expression							
		High	1 (Ref)			1 (Ref)		
		Low	**0.22**	**0.097‐0.51**	**.0004**	**0.20**	**0.086‐0.47**	**.0002**

^a^ Cox regression analysis.

^b^ Mean age.

^c^ According to the Union for International Cancer Control (UICC) staging system.

RFS, recurrence‐free survival; OS, overall survival; nNACT, treated without neoadjuvant chemotherapy; NACT, treated with neoadjuvant chemotherapy; HR, hazard ratio; CI, confidence interval; IGKC, immunoglobulin κ C; bold; Significant *p* values.

**TABLE 2:** Univariate and multivariate analyses of RFS and OS with IGKC expression and clinicopathological characteristics in ESCC patients with/without chemotherapy

			nNACT						NACT					
			Univariate			Multivariate			Univariate			Multivariate		
			HR	95% CI	*p* Value^a^	HR (95% CI)	95% CI	*p* Value^a^	HR (95% CI)	95% CI	*p* Value^a^	HR (95% CI)	95% CI	*p* Value^a^
**RFS**														
	Sex													
		Female	1 (Ref)			1 (Ref)			1 (Ref)			1 (Ref)		
		Male	0.86	0.19‐3.8	.84	0.50	0.095‐2.6	.40	1.4	0.54‐3.5	.51	1.5	0.58‐3.9	.40
	Age													
		<65^b^	1 (Ref)			1 (Ref)			1 (Ref)			1 (Ref)		
		≥65	1.3	0.45‐3.8	.62	1.2	0.37‐3.9	.76	1.1	0.60‐2.0	.77	1.2	0.64‐2.2	.60
	T stage^c^													
		T1, 2	1 (Ref)			1 (Ref)			1 (Ref)			1 (Ref)		
		T3, 4	**11.3**	**3.7‐35**	**<.0001**	**17.7**	**3.5‐88.2**	**.0005**	1.0	0.55‐1.8	.99	1.1	0.59‐2.0	.79
	Lymph node metastasis													
		Negative	1 (Ref)			1 (Ref)			1 (Ref)			1 (Ref)		
		Positive	**3.6**	**1.3‐10**	**.017**	2.2	0.66‐7.2	.20	1.3	0.59‐3.0	.49	1.3	0.59‐3.1	.79
	IGKC expression													
		Low	1 (Ref)			1 (Ref)			1 (Ref)			1 (Ref)		
		High	**0.063**	**0.083‐0.48**	**.0078**	**0.043**	**0.0050‐0.37**	**.0040**	**0.38**	**0.19‐0.75**	**.0054**	**0.35**	**0.18‐0.71**	**.0037**
**OS**														
	Sex													
		Female	1 (Ref)			1 (Ref)			1 (Ref)			1 (Ref)		
		Male	0.58	0.12‐2.7	.48	0.23	0.033‐1.6	.14	1.7	0.39‐7.1	.49	1.6	0.37‐7.1	.52
	Age													
		<65^b^	1 (Ref)			1 (Ref)			1 (Ref)			1 (Ref)		
		≥65	1.1	0.34‐3.7	.85	1.2	0.31‐4.4	.82	1.4	0.62‐3.1	.44	1.7	0.73‐3.9	.22
	T stage^c^													
		T1, 2	1 (Ref)			1 (Ref)			1 (Ref)			1 (Ref)		
		T3, 4	**10.7**	**3.1‐37.3**	**.0002**	**17.8**	**3.4‐94.1**	**.0007**	1.2	0.55‐2.7	.62	1.5	0.63‐3.4	.38
	Lymph node metastasis													
		Negative	1 (Ref)			1 (Ref)			1 (Ref)			1 (Ref)		
		Positive	**3.6**	**1.1‐12.0**	**.033**	2.3	0.52‐9.8	.27	1.6	0.46‐5.2	.48	1.6	0.47‐5.4	.45
	IGKC expression													
		Low	1 (Ref)			1 (Ref)			1 (Ref)			1 (Ref)		
		High	**0.072**	**0.0092‐0.56**	**.012**	**0.046**	**0.0052‐0.41**	**.0059**	**0.38**	**0.15‐0.95**	**.039**	**0.33**	**0.13‐0.84**	**.021**

^a^ Cox regression analysis.

^b^ Mean age.

^c^ According to the Union for International Cancer Control (UICC) staging system.

RFS, recurrence‐free survival; OS, overall survival; nNACT, treated without neoadjuvant chemotherapy; NACT, treated with neoadjuvant chemotherapy; HR, hazard ratio; CI, confidence interval; IGKC, immunoglobulin κ C.

Bold; Significant *p* values.

## 4 DISCUSSION

In the present study, we used scRNA‐seq to understand functions of TIL‐B subtypes in ESCC. Here, we classified TIL‐Bs into 12 subtypes with transcriptomic characteristics. These subtypes included novel subtypes, such as NBC–NF‐κB, MBC–ITGAX and four PB‐subtypes. We identified the MBC–ITGAX subtype, similar to the MBC subtype, and found that the MBC–ITGAX subtype showed an enriched TGFβ GSEA pathway compared with MBCs. TGFβ promotes the activation and proliferation of B cells.^48^ This result indicated that the MBC–ITGAX subtype was a highly activated MBC subtype and had the potential to proliferate.

B cells have various functions depending on their differentiation states, but the details of the functions remain unknown. In NBCs, when antigens bind to their BCR, the NF‐κB pathway is activated and promotes activation and maturation of NBCs.^45^ We found that the NBC–NF‐κB subtype highly expressed NF‐κB‐related genes and unique genes such as *SLAMF1* which encodes T cell stimulating proteins.^38^, ^49^ These results suggest that this subtype was more activated than the NBC subtype and enhanced activation of T cells. The MBC–ITGAX subtype highly expressed genes associated with cell adhesion and co‐stimulation for antigen presentation. Moreover, this subtype showed enrichment of the type I IFN‐related gene set. Type I IFN increases the expression of co‐stimulatory molecules and promotes antigen presentation to T cells.^41^ Taken together, these data suggest that the MBC–ITGAX subtype may promote antigen presentation in response to type I IFN stimulation.

B cells function as antigen‐presentation cells and co‐stimulatory molecules play a critical role in antigen presentation.^42^ CD40, a member of the TNF family, performs important functions as co‐stimulatory molecules in humoral immunity.^50^ The binding of CD40L on T cells to CD40 on B cells contributes to B cell activation via the NF‐κB pathway, proliferation, germinal centre formation and affinity maturation.^51^ CD40 signalling up‐regulates the expression of CD80 and MHC class II and leads to enhanced antigen presentation.^52^ Our analyses showed that NACT enhanced the expression of genes associated with co‐stimulation and CD40 signalling in B cells, except for ASCs, and the MBC–ITGAX subtype up‐regulated TNFα signalling via NF‐κB pathway. Chemotherapy increases TAAs in tumour sites^6^ and enhances the infiltration of CD4^+^ and CD8^+^ T cells in the TIME.^53^ A previous study showed that NACT enhanced Tfh cell functions in pancreatic cancer and Tfh cells treated with NACT promoted B cell maturation.^54^ These data suggest that T cells activated with NACT, including Tfh cells, stimulate the CD40 molecule on B cells and may also promote TIL‐B activations via the NF‐κB pathway and antigen presentation.

NBCs are key cells to initiate humoral immune responses. Our findings revealed a smaller proportion of NBCs in tumour tissues than in normal tissues, consistent with a recent report.^55^ The proportion of NBCs in the TIME treated with NACT was the lowest among the three tissue source types. NBCs with NACT showed up‐regulated expression of activated and enrichment of the NF‐κB‐related pathway associated with B cell activation. Furthermore, NBCs with NACT expressed the suppressing‐receptor gene set at the lowest level among the three tissue source types. The decrease in the proportion of NBCs in metastatic sites was reported to result from B cell activation and differentiation at metastatic regions.^55^ The present data showed the up‐regulation of NF‐κB‐related genes in the NBC–NF‐κB subtype treated with NACT. Furthermore, the NF‐κB‐related genes associated with a favourable prognosis in oesophageal cancer. Taken together, these results suggest that NBCs in the TIME are activated and differentiated via the NF‐κB pathway during chemotherapy and that there is a clinical implication of changes in the NBC–NF‐κB subtype during chemotherapy.

ASCs secrete specific antibodies against TAAs. These antibodies can mediate opsonisation, complement lysis, ADCC by natural killer cells and ADCP by macrophages and granulocytes.^11^ The present data revealed that ASCs in tumour tissues showed up‐regulated expression of *XBP1*, which is a canonical marker of ASCs, compared with expression in normal tissues, and the proportion of ASCs in tumour tissues with NACT was significantly larger than that in normal tissues. Recent studies showed higher numbers of ASCs in tumour sites compared with non‐tumour sites.^56^ Moreover, we showed that ASCs in tumour tissues expressed the homing gene set at a lower level than that in normal tissues, and the gene set expression in ASCs with NACT was lower than that in ASCs without NACT. This result suggests that ASCs in the TIME with NACT were difficult to move out of the tumour areas leading to abundant ASCs in the tumour sites. Additionally, we found that ASCs in tumour tissues with NACT increased the capacity to produce antibodies and DEGs in ASCs during NACT were significantly correlated with a good prognosis in oesophageal cancer^58^ A previous report also showed that IgG produced by local ASCs labeled tumour cells, suggesting that IgG‐dependent anti‐tumoural mechanisms may work in the TIME.^57^ Together, these data suggest that abundant ASCs in the TIME with NACT may play important roles in anti‐tumour immunity.

LNs are important sites for metastasis in solid cancer and the mechanisms underlying metastasis in LNs are complex. We identified seven subtypes of B cells in LNs, similar to the primary tumour, but there were no MBC–ITGAX and PB subtypes in LNs. Instead, we found the GCB‐Ki67 subtype, which was one of the GCB subtypes with a high proliferation potential based on GSEA. Our present data revealed that the proportion of ASCs in metastatic LNs was the highest among three tissue source types, as in the primary tumour. ASCs in the metastatic LN enriched the ASC gene set to the highest levels among tissue source types. Previous studies reported that there were many ASCs in metastatic LNs and the ASCs secreted tumour‐specific antibodies.^46^ B cells except for ASCs enriched the ASC gene set only in the metastatic LN. This suggests that TAAs derived from metastatic tumour cells directly induce activation of B cells and differentiation into ASCs in LNs.

The location and the prognostic significance of ASCs in the TIME remains controversial. Recent studies reported that the presence of ASCs in the TIME was associated with a positive prognosis^8^, ^58^ and high intratumoural immunoglobulin expression was related with longer survival.^59^ First, we examined the location of ASCs in the TIME and ASCs were detected not inside the tumour but in the margin of tumour, in line with a previous report.^60^ Second, we evaluated the prognostic impact of ASCs in ESCC with IGKC expression. The IGKC expression score was significantly higher in recurrence‐free and survival groups. Additionally, high IGKC expression was associated with RFS and OS in ESCC. Moreover, multivariate analyses showed that IGKC expression was an independent favourable prognostic factor of RFS and OS in ESCC patients. Late stages at T3/T4 and LN metastasis were independent unfavourable prognostic factors, as previously reported.^61^, ^62^, ^63^ These data indicate that ASCs in the TIME were related to a good prognosis and that IGKC expression might be a good prognostic marker in ESCC. Additionally, a recent study showed that treatment with NACT increased ASCs in the TIME and influenced prognosis.^64^ Our scRNA‐seq results revealed that the proportion of ASCs and the potential of ASCs in the TIME to secrete antibodies increased with NACT. These data suggest that chemotherapies change the TIME into a favourable environment for anti‐tumour immunity.

In the present study, we observed functional changes in TIL‐Bs of ESCC during chemotherapy. However, this finding has several limitations. First, the number of cells with biopsies versus surgical resected tissues was varied. Second, we did not analyse the samples from the same patients before and after NACT. The TIME is heterogenous and might be different for each patient.

## 5 CONCLUSIONS

Our data revealed that chemotherapy enhanced specific functions, such as co‐stimulation and CD40 signalling, in TIL‐Bs. NBCs were stimulated to activate and differentiate in the ESCC TIME during chemotherapy. There was a large proportion of ASCs secreting amounts of antibodies in the TIME treated with NACT. Changes in ASCs during NACT were associated with a good prognosis in oesophageal cancer. The presence of ASCs in the TIME was associated with a favourable prognosis in ESCC patients. These results suggest that chemotherapy modifies the TIME to be favourable for anti‐tumour immunity and that the dynamic changes in functional heterogeneity of TIL‐Bs may be involved in the anti‐tumour immune response in ESCC. The present data of TIL‐B subtypes with scRNA‐seq provide important findings to understand the role of TIL‐Bs and may contribute to the development of new clinical treatments targeting the TIME.

## CONFLICT OF INTEREST

The authors declare that they have no conflict of interest.

## Supporting information

Supporting Information

Supporting Information

Supporting Information

Supporting Information

Supporting Information

Supporting Information

Supporting Information

Supporting Information

## Data Availability

The data used to support the findings of this study are available from the corresponding author upon reasonable request.
