# Supplementary material for: Single‐cell transcriptome analysis reveals functional changes in tumour‐infiltrating B lymphocytes after chemotherapy in oesophageal squamous cell carcinoma
Source: Clin Transl Med. 2023 Jan 17;13(1):e1181. doi: 10.1002/ctm2.1181 (PMC9845121; doi:10.1002/ctm2.1181)
Supplement: Supplementary file 1 — Supporting Information [file CTM2-13-e1181-s007.docx]

**Supplementary Data**

**Supplementary Table legends**

**Supplementary Table S1. Characteristics of patients with esophageal squamous cell carcinoma in this study.**

**Supplementary Table S2. Antibody targets with CODEX.**

**Supplementary Table S3. Summary of CODEX multiple staining.**

**Supplementary Table S4. Components of each gene set in our study.**

**Supplementary Table S5. The top genes upregulated after chemotherapy in ASCs.**

**Supplementary Table S6. Clinicopathological characteristics in patients of esophageal squamous cell carcinoma with/without neoadjuvant chemotherapy for immunohistochemistry.**

| **Supplementary Table S1. Characteristics of patients with esophageal squamous cell carcinoma in this study.** | | | | | | | |
| --- | --- | --- | --- | --- | --- | --- | --- |
| Sample ID | Age | Sex | TNM (UICC)^a^ | Stage (UICC)^a^ | Collection | NACT | Histology |
| ESC04 | 58 | Male | ypT3N2M0 | IIIB | Surgery | FP | SCC |
| ESC07 | 52 | Male | pT2N1M0 | IIIA | Surgery | none | SCC |
| ESC09 | 80 | Female | ypT1bN0M0 | IB | Surgery | FP | SCC |
| ESC15 | 71 | Male | ypT3N3M0 | IVA | Surgery | FP+DCF | SCC |
| ESC17 | 62 | Male | pT1aN2M0 | IIIA | Surgery | none | SCC |
| ESC18 | 74 | Female | pT1bN0M0 | IB | Surgery | none | SCC |
| ESC19 | 72 | Male | cT2N0M0 | II | Endoscopic biopsy | none | SCC |
| ESC20 | 58 | Male | ypT3N0M0 | IIB | Surgery | FP | SCC |
| ESC21 | 69 | Male | cT4bN2M1 | IVB | Endoscopic biopsy | none | SCC |
| ESC22 | 70 | Female | ypT4aN2M0 | IVA | Surgery | FP | SCC |
| ESN04 | 58 | Male | -- | -- | Surgery | -- | Normal tissue |
| ESN06 | 41 | Female | -- | -- | Surgery | -- | Normal tissue |
| ESN08 | 54 | Male | -- | -- | Surgery | -- | Normal tissue |
| ESN11 | 70 | Female | -- | -- | Surgery | -- | Normal tissue |
| ESN12 | 61 | Male | -- | -- | Surgery | -- | Normal tissue |
| ESN13 | 65 | Male | -- | -- | Surgery | -- | Normal tissue |
| ESN15 | 71 | Male | -- | -- | Surgery | -- | Normal tissue |
| LN07 | 52 | Male | -- | -- | Surgery | none | Normal LN |
| LN09 | 80 | Female | -- | -- | Surgery | FP | Normal LN |
| LN12 | 61 | Male | -- | -- | Surgery | FP | Normal LN |
| LN26 | 84 | Male | -- | -- | Surgery | FP | Metastatic LN |
| LN45 | 89 | Female | -- | -- | Surgery | none | Normal LN |
| LN49 | 62 | Female | -- | -- | Surgery | none | Normal LN |
| ^a^According to the Union for International Cancer Control (UICC) staging system. | | | | | | | |
| NACT, neoadjuvant chemotherapy; FP, fluorouracil + cisplatin; DCF, docetaxel + cisplatin + fluorouracil; | | | | | | | |
| SCC, squamous cell carcinoma; LN, lymph node. | | | | | | | |

| **Supplementary Table S2. Antibody targets with CODEX.** | | | | | |
| --- | --- | --- | --- | --- | --- |
| Antibody-target | Company | Catalog # | CODEX barcode | Clone | Fluorophore |
| CD3e | Akoya Biosciences | 4450030 | BX045 | EP449E | Cy5 |
| CD8 | Akoya Biosciences | 4250012 | BX026 | C8/144B | Atto550 |
| CD20 | Akoya Biosciences | 4450018 | BX007 | L26 | Alexa Fluor™ 750 |
| Pan-Cytokeratin | Akoya Biosciences | 4450020 | BX019 | AE-1/AE-3 | Alexa Fluor™ 750 |
| Ki67 | Akoya Biosciences | 4250019 | BX047 | B56 | Atto550 |

| **Supplementary Table S3. Summary of CODEX multiple staining.** | | | | | | | | | | | | | | | | | |
| --- | --- | --- | --- | --- | --- | --- | --- | --- | --- | --- | --- | --- | --- | --- | --- | --- | --- |
| Reg | Cyc | Ch | Marker | Exposure | Min | Median | 95% | Max | Mean | Std Dev^a^ | Threshold | Area | Signal µ | Signal σ | Noise µ | Noise σ | SNR^b^ |
| 2 | 1 | 1 | DAPI1 | 10 ms | 0 | 300 | 19978 | 65220 | 3177.4 | 8326.9 | 13056 | 7.90% | 27370.8 | 13134.9 | 1089.4 | 2357.1 | 11.6 |
| 2 | 1 | 2 | Blank | 350 ms | 0 | 5585 | 22585 | 65535 | 8032.8 | 9028.8 | 22528 | 4.80% | 40835.5 | 12818.7 | 6386.7 | 4544.8 | 9 |
| 2 | 1 | 3 | Blank | 500 ms | 0 | 980 | 2873 | 65535 | 1649.1 | 4339.3 | 17408 | 1.30% | 35717.5 | 12961.8 | 1203.2 | 1219.4 | 29.3 |
| 2 | 1 | 4 | Blank | 500 ms | 0 | 105 | 320 | 65535 | 134.7 | 137.9 | 256 | 0.90% | 921 | 724.8 | 127.5 | 92.7 | 9.9 |
| 2 | 2 | 1 | DAPI2 | 10 ms | 0 | 262 | 12249 | 65535 | 2098.5 | 5640.8 | 9216 | 6.70% | 19422.4 | 10516.5 | 850.8 | 1724.7 | 11.3 |
| 2 | 2 | 2 | Ki67 | 350 ms | 0 | 1 | 1009 | 65535 | 421.9 | 2890.5 | 12544 | 0.80% | 27137.1 | 13677.9 | 202.7 | 991.8 | 27.4 |
| 2 | 2 | 3 | CD107a | 500 ms | 0 | 418 | 13649 | 65534 | 2771.7 | 6963.7 | 12288 | 5.50% | 26267.8 | 13876.9 | 1412.2 | 2522.7 | 10.4 |
| 2 | 2 | 4 | CD20 | 500 ms | 0 | 10 | 210 | 65535 | 72.5 | 713.5 | 6912 | 0.10% | 15617.9 | 10702.4 | 53 | 246 | 63.5 |
| 2 | 3 | 1 | DAPI3 | 10 ms | 0 | 243 | 11702 | 65535 | 2004.2 | 5422.7 | 8704 | 6.80% | 18500.2 | 10203.7 | 800.6 | 1628.5 | 11.4 |
| 2 | 3 | 2 | CD8 | 350 ms | 0 | 10 | 1089 | 65535 | 412.3 | 2789.6 | 12544 | 0.70% | 28161.8 | 14934.1 | 218.6 | 930.9 | 30.3 |
| 2 | 3 | 3 | CD3e | 500 ms | 0 | 28 | 620 | 65535 | 171.1 | 697.8 | 2048 | 1.20% | 4878.9 | 3479.6 | 114.4 | 276.6 | 17.6 |
| 2 | 3 | 4 | PanCK | 500 ms | 0 | 83 | 11778 | 65535 | 2080.6 | 5431.9 | 8704 | 7.20% | 17874.9 | 10001.5 | 860.1 | 1813.8 | 9.9 |
| 2 | 4 | 1 | DAPI4 | 10 ms | 0 | 230 | 12294 | 65535 | 2077.9 | 5681.5 | 9216 | 6.70% | 19505.4 | 10606.6 | 818.7 | 1718.8 | 11.3 |
| 2 | 4 | 2 | Blank | 350 ms | 0 | 4107 | 13131 | 65535 | 5682.7 | 7261.8 | 23808 | 2.40% | 45562.6 | 11369.8 | 4698.2 | 3257.9 | 14 |
| 2 | 4 | 3 | Blank | 500 ms | 0 | 877 | 2167 | 65535 | 1332 | 3223.4 | 13312 | 1.20% | 27370.3 | 11580.9 | 1025.7 | 929.5 | 29.4 |
| 2 | 4 | 4 | Blank | 500 ms | 0 | 110 | 335 | 65535 | 141.3 | 173.1 | 512 | 0.40% | 1501.9 | 1740 | 135.9 | 104.1 | 14.4 |
| ^a^Std Dev, standard deviation; ^b^SNR, the signal to noise ratio. | | | | | | | | | | | | | | | | | |

| **Supplementary Table S4. Components of each gene set in our study.** | | | | | | | | |
| --- | --- | --- | --- | --- | --- | --- | --- | --- |
| Gene set | Source | Genes |  |  |  |  |  |  |
| Type I IFN | Ye X, et al. 2022 | IFIT1 | IFIT2 | IFIT3 | IRF7 | ISG15 |  |  |
| co-stimulation | Choi IK, et al. 2018 | CD40 | CD80 | CD86 | ICAM1 | CD70 | TNFSF4 | TNFSF9 |
| CD40 signaling | Holmes AB, et al. 2020 | CD40 | TRAF1 | ICAM1 | CFLAR | BCL2A1 | BCL2L1 | CD80 |
|  |  | STAT5A | CFLAR | CD86 | MIR155HG | EBI3 | CD58 | CCL22 |
| suppressing-receptor | Nitschke L. 2005 | CD22 | CD72 | FCGR2B |  |  |  |  |
| homing | Nguyen DC, et al. 2021 | CXCR4 | S1PR1 | ITGAL | CXCR3 | ZFP36L1 |  |  |
| antibody-secreting | Yu J, et al. 2020 | IGHG1 | IGHG2 | IGHG3 | IGHG4 |  |  |  |
| Naive | Zhao J, et al. 2020 | IGHD | IGHM | TCL1A |  |  |  |  |
| ASC | Wieland A, et al.2021 | XBP1 | PRDM1 | MZB1 |  |  |  |  |

| **Supplementary Table S5. The top genes upregulated after chemotherapy in ASCs** | | | | | |
| --- | --- | --- | --- | --- | --- |
| Genes | p_val | avg_log2FC | pct.1 | pct.2 | p_val_adj |
| XIST | 4.81E-143 | 1.727609568 | 0.56 | 0 | 1.44E-138 |
| S100A2 | 3.28E-50 | 0.307668132 | 0.169 | 0.486 | 9.81E-46 |
| MTRNR2L8 | 4.17E-44 | 0.749729836 | 0.354 | 0.089 | 1.25E-39 |
| MTRNR2L12 | 4.04E-40 | 1.069136648 | 0.683 | 0.393 | 1.21E-35 |
| MGP | 5.22E-27 | 1.021666462 | 0.159 | 0.012 | 1.56E-22 |
| IGHG3 | 9.61E-27 | 0.703821662 | 0.874 | 0.793 | 2.88E-22 |
| IGKC | 8.95E-26 | 0.463329703 | 0.992 | 0.936 | 2.68E-21 |
| HSPA1A | 1.25E-25 | 0.660142456 | 0.732 | 0.51 | 3.76E-21 |

| **Supplementary Table S6. Clinicopathological characteristics in patients of esophageal squamous cell carcinoma with/without neoadjuvant chemotherapy for immunohistochemistry.** | | | | | | | | |
| --- | --- | --- | --- | --- | --- | --- | --- | --- |
|  |  | **nNACT^a^** |  |  |  | **NACT^a^** |  |  |
|  |  | High | Low | ***p^b^*** |  | High | Low | ***p^b^*** |
|  |  | No. (%) |  |  |  | No. (%) |  |  |
| All cases | 166 | 40 | 40 |  |  | 35 | 51 |  |
| Sex |  |  |  | 0.74 |  |  |  | 0.77 |
|  | Male | 34 (85.0) | 36 (90.0) |  |  | 30 (85.7) | 42 (82.4) |  |
|  | Female | 6 (15.0) | 4 (10.0) |  |  | 5 (14.3) | 9 (17.6) |  |
| Age |  |  |  | 0.26 |  |  |  | 0.67 |
|  | <65^c^ | 17 (42.5) | 23 (57.5) |  |  | 16 (45.7) | 26 (51.0) |  |
|  | ≥65 | 23 (57.5) | 17 (42.5) |  |  | 19 (54.3) | 25 (49.0) |  |
| T stage^d^ |  |  |  | 0.26 |  |  |  | 0.39 |
|  | T1/T2 | 38 (95.0) | 34 (85.0) |  |  | 15 (42.9) | 27 (52.9) |  |
|  | T3/T4 | 2 (5.0) | 6 (15.0) |  |  | 20 (57.1) | 24 (47.1) |  |
| Stage (UICC)^d^ |  |  |  | 0.40 |  |  |  | 0.31 |
|  | I/II | 34 (85.0) | 30 (75.0) |  |  | 6 (17.1) | 14 (27.5) |  |
|  | III/IV | 6 (15.0) | 10 (25.0) |  |  | 29 (82.9) | 37 (72.5) |  |
| Lymph node metastasis  (negative vs positive) |  |  |  | 0.46 |  |  |  | 0.78 |
|  | Negative | 30 (75.0) | 26 (65.0) |  |  | 6 (17.1) | 11 (21.6) |  |
|  | Positive | 10 (25.0) | 14 (35.0) |  |  | 29 (82.9) | 40 (78.4) |  |
| ^a^nNACT; treated without neoadjuvant chemotherapy, NACT; treated with neoadjuvant chemotherapy. | | | | | | | | |
| ^b^Chi-square test or Fisher’s exact test. | | | | | | | | |
| ^c^Mean age. | | | | | | | | |
| ^d^According to the Union for International Cancer Control (UICC) staging system. | | | | | | | | |

**Supplementary Figure legends**

**Supplementary Figure S1. Single cell data integration and profiles.**

A, B) The UMAP plots were colored by tissue histology (normal or tumor) (A) and treatment with/without NACT (NACT and nNACT, respectively) (B).

C) The UMAP plots showed major cell types by the tissue source type (normal tissues (N), tumors without NACT (T-nNACT) and tumors with NACT (T-NACT)).

**Supplementary Figure S2. Known major B cell subtypes. The expression of immunoglobulin isotypes and MHC class I/II in TIL-Bs and characteristics in the MBC-ITGAX subtype. CODEX multiple staining in the TIME.**

A–C) UMAP plots of B cells were colored by the sample origin (A), tissue histology (B) and treatment with/without NACT (C).

D) UMAP plots showed the known major subtypes of TIL-Bs.

E) Heatmap indicated canonical genes of B cells in each major subtype.

F, G) UMAP plots showed the expression levels of genes associated with immunoglobulin isotypes (IgA, IgD, IgE, IgG1 and IgM) (F) and with MHC class I/II (MHC class I: *HLA-A*; MHC class II: *HLA-DRA*) (G).

H) Gene Set Enrichment Analysis (GSEA) results showed B cell fluarix up and TGFβ signaling pathway enriched in the MBC-ITGAX subtype.

I) CODEX multiple staining images were shown. Individual images of DAPI, CD3e, CD8, CD20, PanCK and Ki67 staining were shown (from left to right). Scale bars, 1000μm.

**Supplementary Figure S3. Detailed functional analyses in each TIL-B subtype.**

A) Heatmap showed the expression of B cell function–associated genes in each subtype.

B) Heatmap showed the expression of HSP–related genes in ASCs.

**Supplementary Figure S4. Major cell types in lymph nodes.**

A) UMAP plot showed 16 clusters derived from 5 regional lymph nodes (LNs) without metastasis and 1 regional LN with metastasis.

B–D) UMAP plot was colored by sample origin (B), tissue histology (with metastasis and without metastasis (nMetastasis)) (C) and treatment with/without NACT (D).

E) UMAP plots showed the expression levels of canonical marker genes and characteristic genes.

F) Heatmap of the canonical marker genes and characteristic genes in each cluster.

G) UMAP plot indicated three cell types on the basis of canonical marker genes and characteristic genes.

H) UMAP plot in the LN26 sample showed the expression levels of squamous epithelial marker (*KRT5*, *KRT19*, *EPCAM* and *SOX4*).

I) The number of the major cell types by sample origin.

**Supplementary Figure S5. B cell subtypes in LNs and characteristics in the GCB-Ki67 subtype.**

A–C) UMAP plots of B cells in LNs were colored by the sample origin (A), tissue histology (B) and treatment with/without NACT (C).

D) GSEA results showed IgG-Memory/Plasma cell down, Naive/GC B cell down and E2F pathway enriched in the GCB-Ki67 subtype.

**Supplementary Figure S6. Changes in expression levels of *TNF* and *HLA-DQA2* with NACT.**

A, B) Violin plots showed the expression of *TNF* (A) and *HLA-DQA2* (B) between nM-nNACT and nM-NACT. Significance of *TNF* and *HLA-DQA2* expressions (p-value) between tissue source types was determined by Wilcoxon rank-sum test. Boxplots included centerline, median; box limits, upper and lower quartiles; whiskers at most 1.5× the interquartile range past upper and lower quartiles. ^****^*p* < 0.0001.

**Supplementary Figure S7. Representative images showing IGKC immunostaining intensity (score 0~+3) (magnification ×400). Scale bars, 50μm.**
