# Supplementary figures and images for: Single‐cell transcriptome analysis reveals functional changes in tumour‐infiltrating B lymphocytes after chemotherapy in oesophageal squamous cell carcinoma
Source: Clin Transl Med. 2023 Jan 17;13(1):e1181. doi: 10.1002/ctm2.1181 (PMC9845121; doi:10.1002/ctm2.1181)

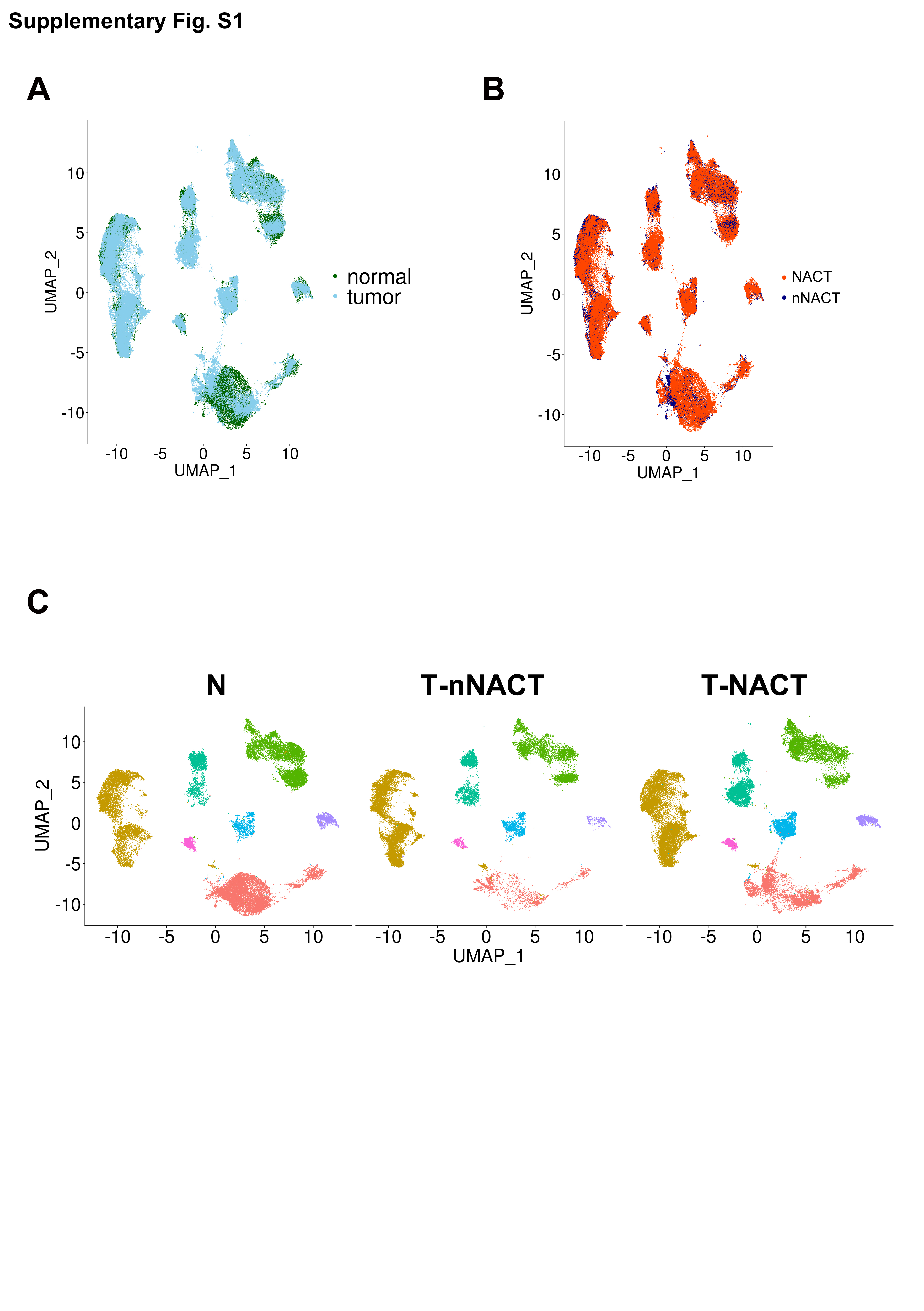

Supplement: Supplementary file 2 — Supporting Information [file CTM2-13-e1181-s001.jpg]

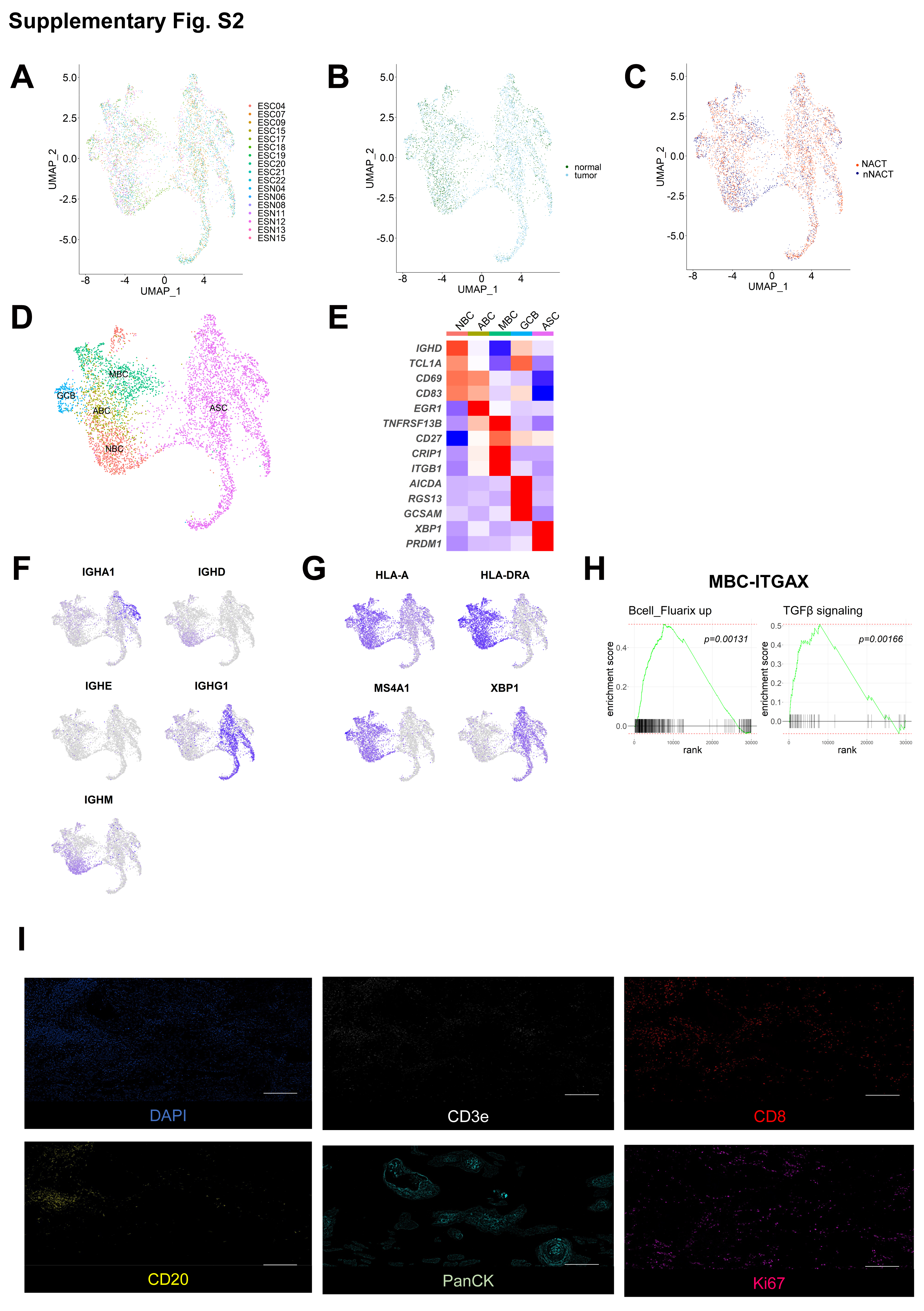

Supplement: Supplementary file 3 — Supporting Information [file CTM2-13-e1181-s003.jpg]

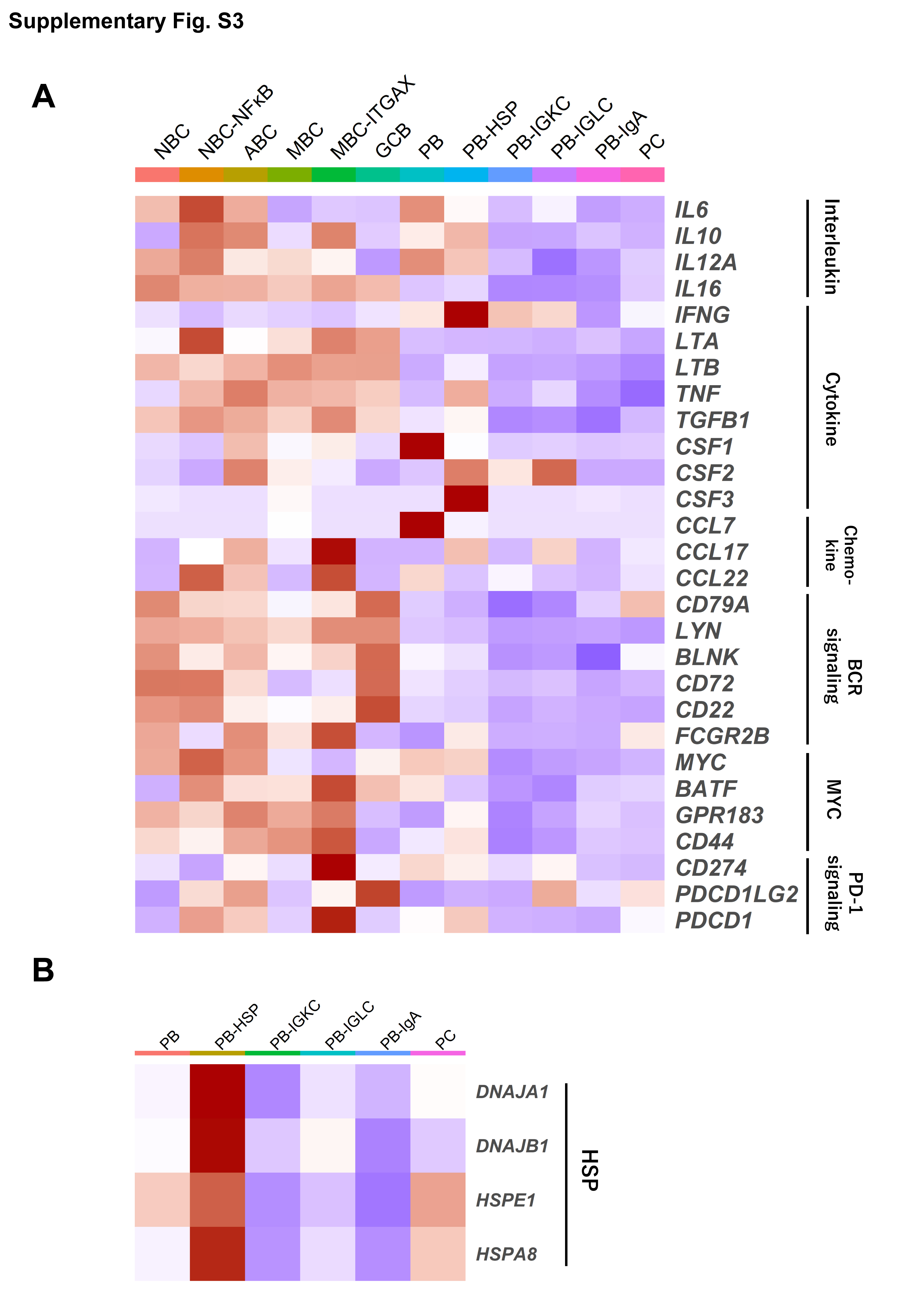

Supplement: Supplementary file 4 — Supporting Information [file CTM2-13-e1181-s006.jpg]

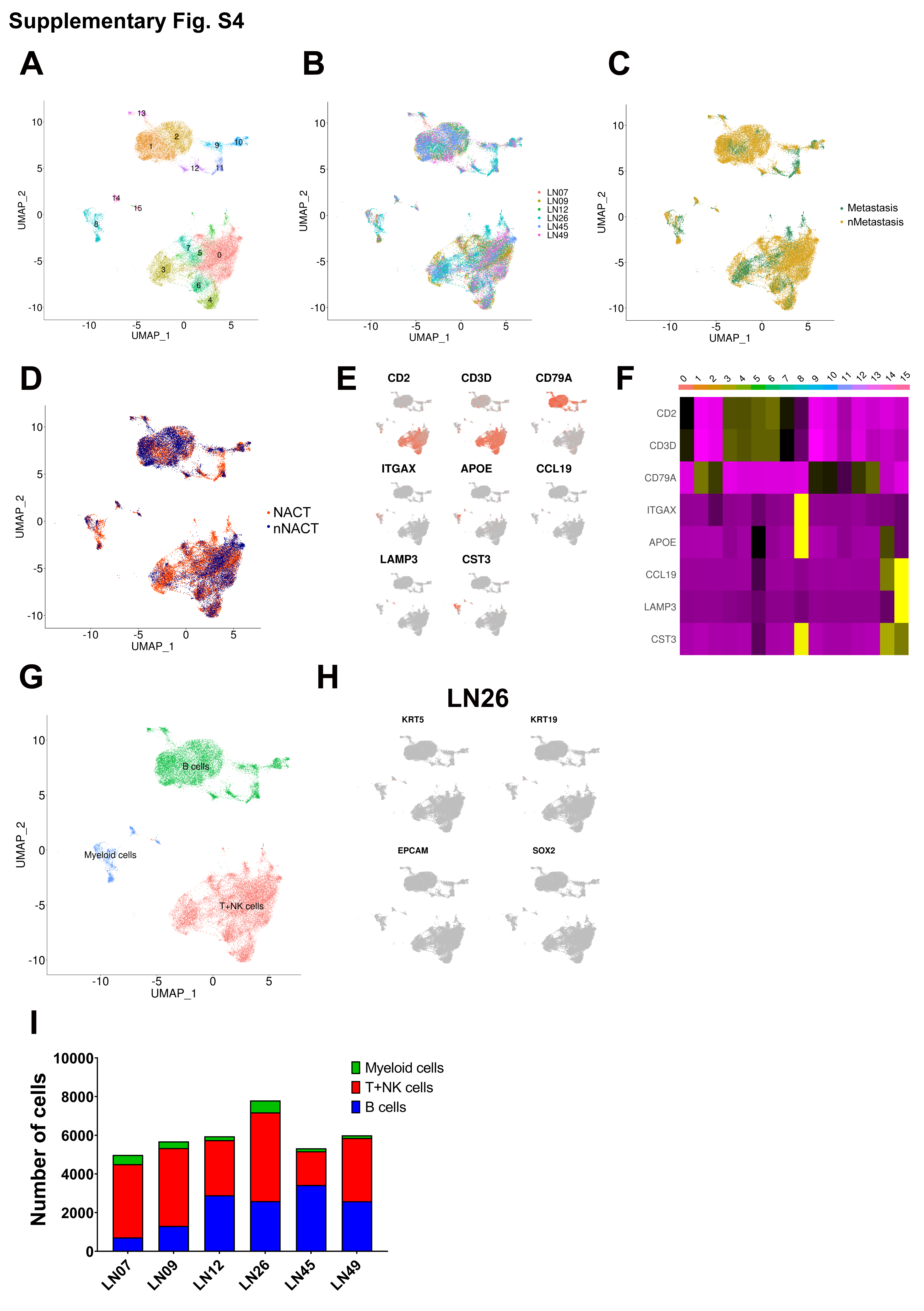

Supplement: Supplementary file 5 — Supporting Information [file CTM2-13-e1181-s004.jpg]

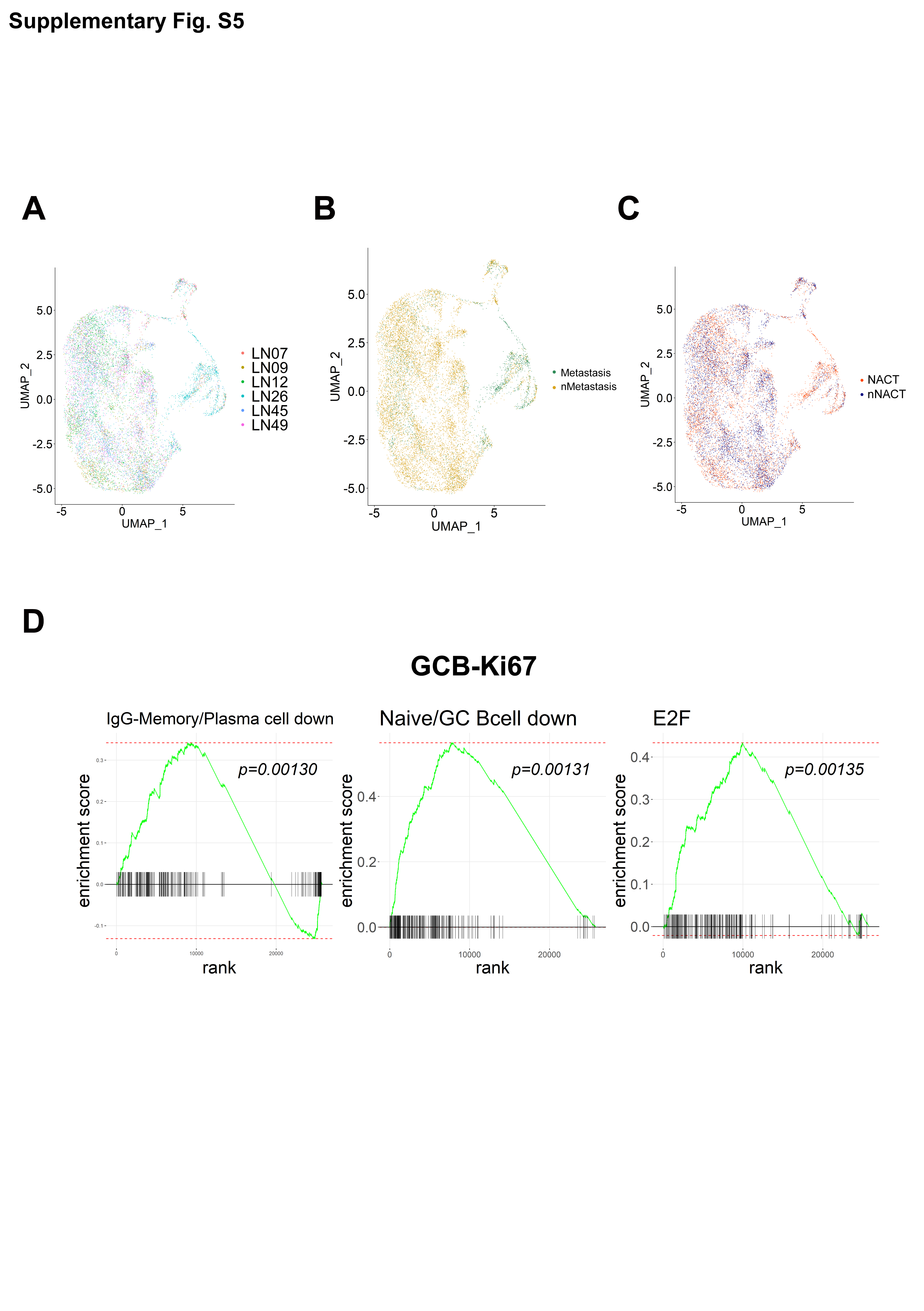

Supplement: Supplementary file 6 — Supporting Information [file CTM2-13-e1181-s005.jpg]

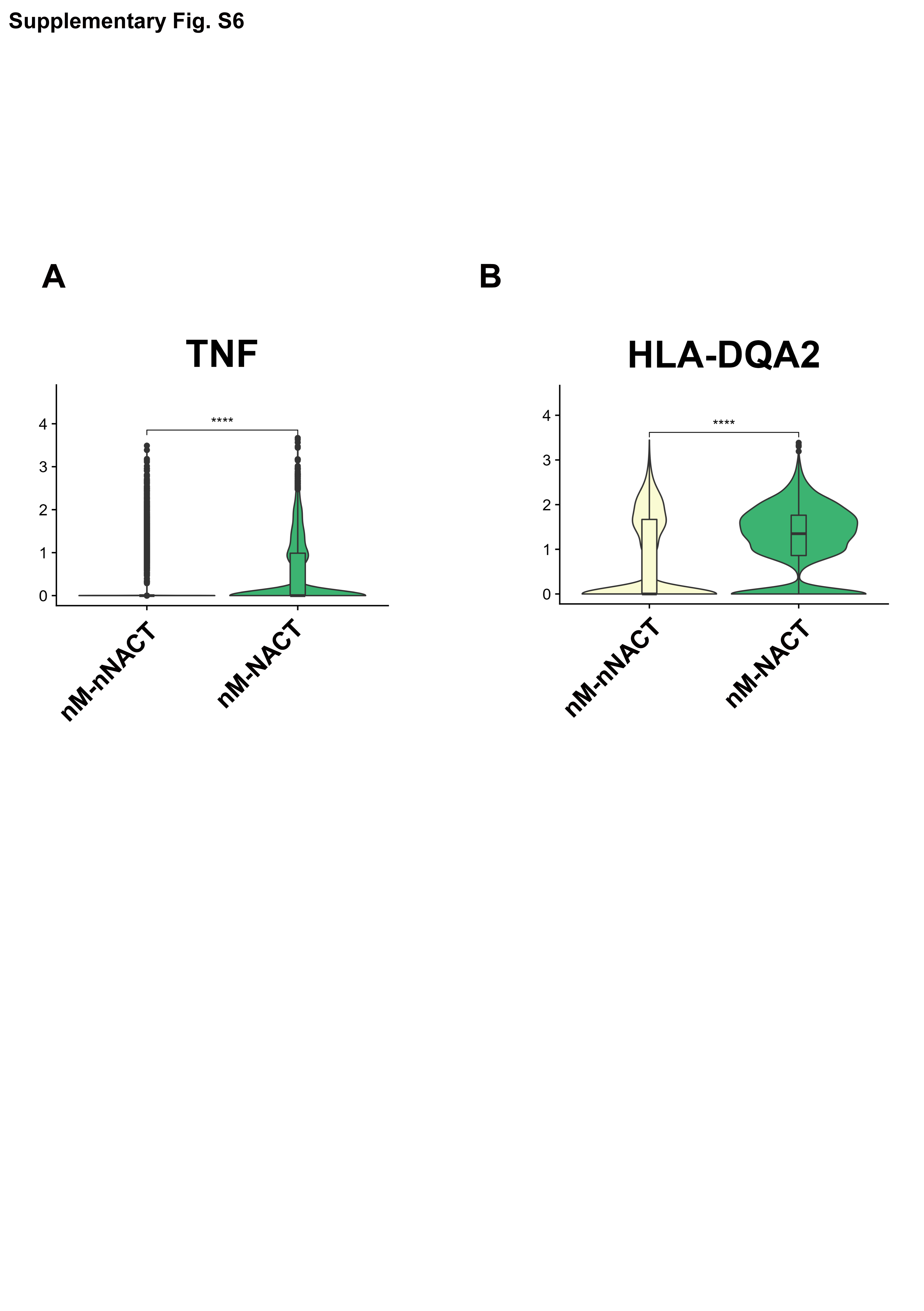

Supplement: Supplementary file 7 — Supporting Information [file CTM2-13-e1181-s002.jpg]

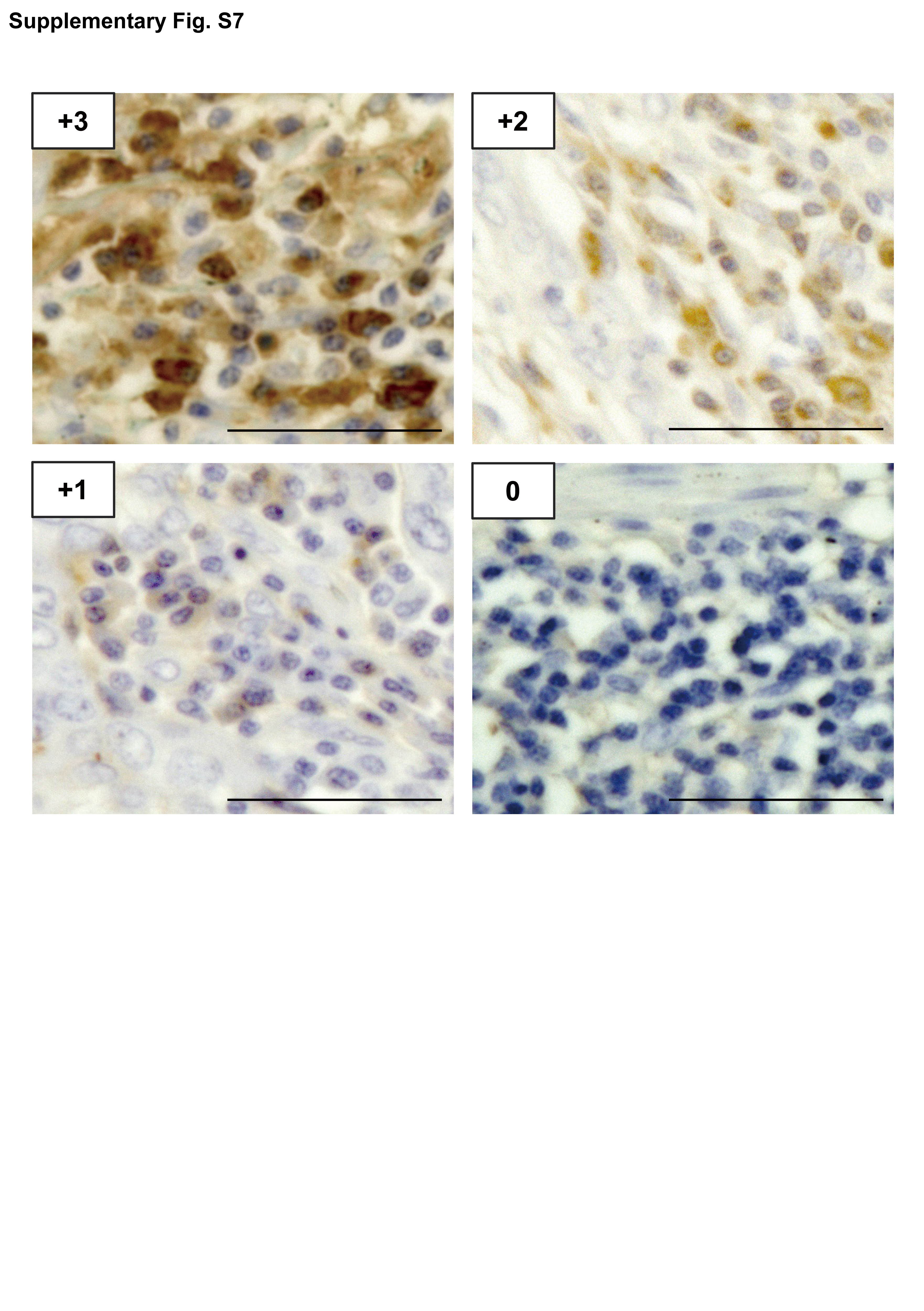

Supplement: Supplementary file 8 — Supporting Information [file CTM2-13-e1181-s008.jpg]
